# Comprehensive analysis of the *Xya riparia* genome uncovers the dominance of DNA transposons, LTR/Gypsy elements, and their evolutionary dynamics

**DOI:** 10.1186/s12864-024-10596-5

**Published:** 2024-07-12

**Authors:** Hashim Khan, Huang Yuan, Xuanzeng Liu, Yimeng Nie, Muhammad Majid

**Affiliations:** College of Life Sciences, Shaanxi Normal University, Xian, China

**Keywords:** Age analysis, Orthoptera, Pygmy mole crickets, Transposable elements

## Abstract

Transposable elements (TEs) are DNA sequences that can move or replicate within a genome, and their study has become increasingly important in understanding genome evolution and function. The Tridactylidae family, including *Xya riparia* (pygmy mole cricket), harbors a variety of transposable elements (TEs) that have been insufficiently investigated. Further research is required to fully understand their diversity and evolutionary characteristics. Hence, we conducted a comprehensive repeatome analysis of *X. riparia* species using the chromosome-level assembled genome. The study aimed to comprehensively analyze the abundance, distribution, and age of transposable elements (TEs) in the genome. The results indicated that the genome was 1.67 Gb, with 731.63 Mb of repetitive sequences, comprising 27% of Class II (443.25 Mb), 16% of Class I (268.45 Mb), and 1% of unknown TEs (19.92 Mb). The study found that DNA transposons dominate the genome, accounting for approximately 60% of the total repeat size, with retrotransposons and unknown elements accounting for 37% and 3% of the genome, respectively. The members of the Gypsy superfamily were the most abundant amongst retrotransposons, accounting for 63% of them. The transposable superfamilies (LTR/Gypsy, DNA/nMITE, DNA/hAT, and DNA/Helitron) collectively constituted almost 70% of the total repeat size of all six chromosomes. The study further unveiled a significant linear correlation (Pearson correlation: *r* = 0.99, p-value = 0.00003) between the size of the chromosomes and the repetitive sequences. The average age of DNA transposon and retrotransposon insertions ranges from 25 My (million years) to 5 My. The satellitome analysis discovered 13 satellite DNA families that comprise about 0.15% of the entire genome. In addition, the transcriptional analysis of TEs found that DNA transposons were more transcriptionally active than retrotransposons. Overall, the study suggests that the genome of *X. riparia* is complex, characterized by a substantial portion of repetitive elements. These findings not only enhance our understanding of TE evolution within the Tridactylidae family but also provide a foundation for future investigations into the genomic intricacies of related species.

**Supplementary Information:**

The online version contains supplementary material available at 10.1186/s12864-024-10596-5.

## Background

Pygmy mole crickets, also known as sand grasshoppers, belong to the Tridactylidae family. This family is the most well-known among tridactyloids, primarily because of their global presence and the significant number of extant and fossil species they encompass [1]. According to the Integrated Taxonomic Information System (ITIS), there are currently 147 recognized species in the family Tridactylidae, which includes the pygmy mole crickets(https://www.itis.gov/servlet/SingleRpt/SingleRpt? search_topic=TSN&search_value=155170#null, accessed online, March 2023). Pygmy mole crickets are found across the globe, inhabiting various regions except the poles. Unlike other orthopteran species in the suborder Caelifera, which typically have larger body sizes, pygmy mole crickets have a significantly smaller body length of 3.8–10 mm [2]. The evolutionary history of pygmy mole crickets is still poorly understood, but recent molecular studies have provided insights into their phylogenetic relationships and divergence times. That suggests that the family *Tridactylidae* is a relatively old lineage, with origins dating back to the early Cretaceous period [3, 4]. Similarly, genomic and transcriptomic analysis of the family *Tridactylidae* is rarely reported in the literature. But in our recent work, we reported a chromosome-level assembly of *X. riparia* species (Pygmy mole cricket) with a genome size of 1.67 GB [5]. Compared to other orthopteran species, the genome size of the pygmy mole cricket was found to be relatively lower. Among the orthopteran species, the grasshoppers of the Acrididae family have the largest genomes. The largest insect genomes recorded were *Podisma pedestris* (1 C = 16.93 pg) [6], *Bryodemella holdereri* (1 C = 18.64 pg) [7] and *Stethophyma grossum* (1 C = 18.48) [8]. The large size of these genomes has been suggested to be due to satellite DNAs and transposable elements [9–12]. However, the lack of correlation between chromosome number and genome size makes complete genome duplications less likely. Despite ensiferans typically having higher chromosome numbers, they generally possess smaller genomes compared to caeliferans [13]. Previously, the ensiferan species *Deracantha onos* held the record for genome size in the Orthoptera group, with a genome size of 1 C = 19.60 pg [14]. However, a recent report revealed that *Bryodemella tuberculata* (Caelifera: Acrididae) now holds the record for the largest genome size among all insects, measuring 1 C = 21.96 pg [15].

Many studies reported a positive correlation between genome size and transposable elements, which means that larger genomes tend to have more transposable elements than smaller genomes. This is because transposable elements can make up a significant proportion of a genome, and their proliferation can contribute to genome expansion [16–19]. TE content varies significantly in eukaryotes and plays an important role in determining the genome size and organization. Some species, such as humans, maize, wheat, and lungfish, have TE-rich genomes, with TE content ranging from approximately 45%, 85%, 85%, and 90%, respectively [20–22]. On the other hand, species like *Drosophila melanogaster* and *Arabidopsis thaliana* have TE-poor genomes, with repeatome constituting approximately 10–12 and 15% of their genomes [23, 24]. These variations in TE content are also observed within animal clades, including insects, where TE content ranges from as low as 2% in the Antarctic midge [25] to 65% in *Locusta migratoria* [26, 27] and as high as 75% in morabine grasshoppers of the *Vandiemenella viatica* species [28].

*X. riparia* are typically found in moist habitats such as rice paddies, marshes, and streamsides across various regions of China. *X. riparia* is known for its unique characteristics, including its small size, cryptic coloration, and specialized adaptations for living in aquatic environments. These insects play ecological roles in their habitats, contributing to nutrient cycling and serving as prey for various predators. The genome size and genomic and transcriptomic analyses of the *X. riparia* species have been reported, but it lacks an in-depth analysis of mobile elements within the genome. In the present study, we aimed to conduct an in-depth analysis of the mobilome of *X. riparia* at a genome-wide scale. To achieve this, we utilized a chromosome-level genome assembly of *X. riparia* species belonging to the Tridactylidae family. The TE analysis conducted in this study has provided improved accuracy and resolution of TE annotation and classification. Studying transposable elements and repetitive sequences of Tridactylidae family species is essential for comprehending the evolution and function of their in the genomes.

## Results

### Whole-genome TEs characterization and their distribution across chromosomes

**Fig. 1:**
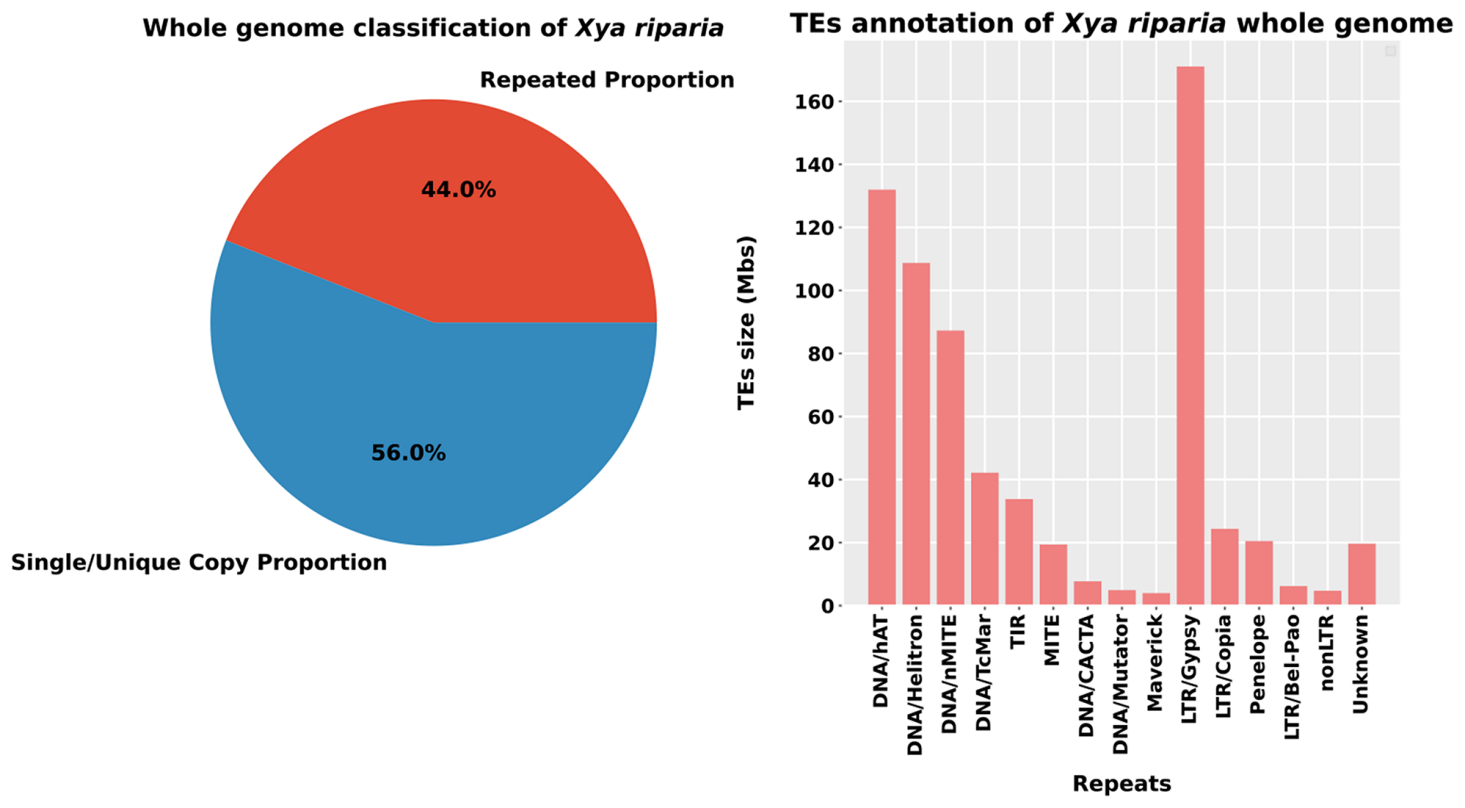
The complete genome analysis of transposable elements (TEs). The genome was divided into two parts - one representing the proportion of repetitive sequences and the other representing coding or single-copy sequences (**shown on the left**). The right-side graph shows the total size (in Mb) of each superfamily in the genome. The x-axis indicates the name of each family, while the y-axis represents the size of the TEs in megabase pairs (Mbp) (**right**)

We conducted a comprehensive repeatome analysis of the *X. riparia* genome assembly, which revealed the presence of 731.63 Mb of repetitive sequences. Among these repetitive sequences, 443.25 Mb (27%) were classified as DNA transposons (Class II), 268.45 Mb (16%) were classified as retroelements (Class I), and 19.92 Mb (1%) were classified as unknown TEs. Of all the repetitive sequences identified, members of LTR/Gypsy superfamily (Class I) accounted for 171.05 Mb of the assembly, while the DNA/hAT, DNA/Helitron, and Class II/nMITE repeats in Class II accounted for 131.95 Mb, 108.71 Mb, and 87.26 Mb, respectively. These four types of repetitive sequences were found to be the most dominant among all the repetitive sequences. Other major TEs included DNA transposon DNA/TcMar, TIR, and MITE, which accounted for 42.16 Mb, 33.79 Mb, and 19.38 Mb, respectively. Conversely, retrotransposons included LTR/Copia and Penelope, which accounted for 24.35 Mb and 20.46 Mb, respectively. The genome of *X. riparia* was mainly composed of DNA transposons, making up approximately 60% of the total repeat size. Retrotransposons and unknown elements accounted for 37% and 3% of the total repeat size, respectively. Specifically, among the retrotransposons, the LTR/Gypsy element constituted almost 64% of the total size. These collective findings suggest that the genome of *X. riparia* is complex and contains a substantial amount of transposable elements (see Fig. 1).

**Fig. 2:**
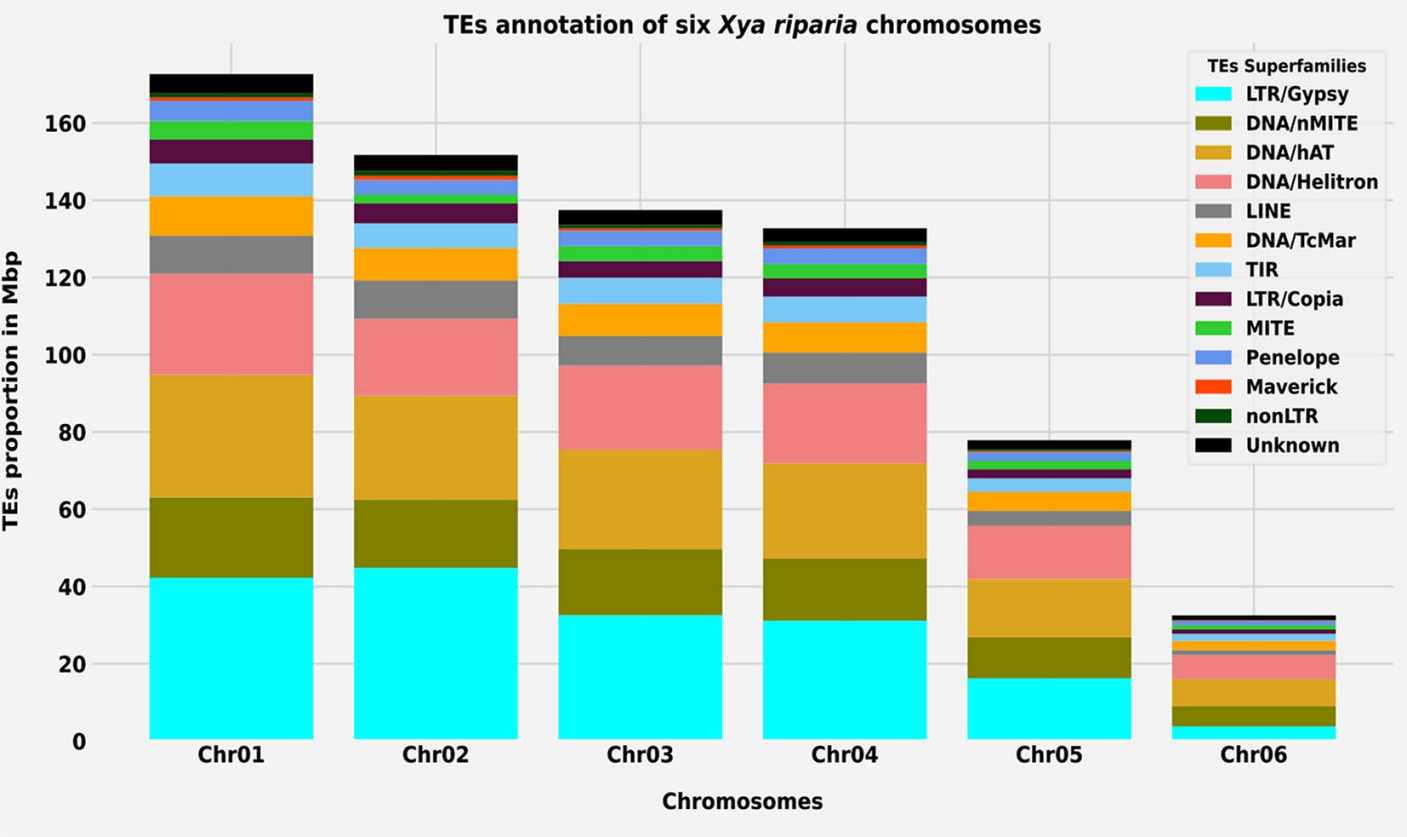
Superfamilies-level Transposable elements annotation of six *X. riparia* chromosomes. Abundance and distribution comparison of transposable elements across six chromosomes

We employed a comprehensive approach to quantifying the abundance and distribution of transposable elements in the *X. riparia* genome. Our findings indicate that these elements are distributed across all six chromosomes, with some variations in their distribution patterns. Notably, the LTR/Gypsy elements were found to be the most abundant on chromosome 2, the largest in size, and the least abundant on chromosome 6 (Fig. 2). The distribution of DNA/Helitron elements also varied among the chromosomes, with the highest concentration on chromosome 1 and the second-highest concentration on chromosome 4. Additionally, the proportion of DNA/Helitron elements was lower on chromosome 2 than on chromosome 3. It is worth noting that the four transposable superfamilies (i.e., LTR/Gypsy, DNA/nMITE, DNA/hAT, and DNA/Helitron) collectively constituted almost 70% of the total repeat size of all six chromosomes (Fig. 2). In addition, we noted that the size of the repetitive sequences on the chromosomes was directly correlated with the size of the chromosomes themselves. We determined the total size and distribution of repetitive sequences by analyzing the sequence composition of all the chromosomes. Our analysis revealed a significant linear correlation (Pearson’s correlation: rs = 0.99, *p*-value = 0.00003) between the size of the chromosomes and the repetitive sequences (see Figure S1 Table S1).

### Repeat landscape of class II and class I elements and insertion timing of major superfamilies of TEs

**Fig. 3:**
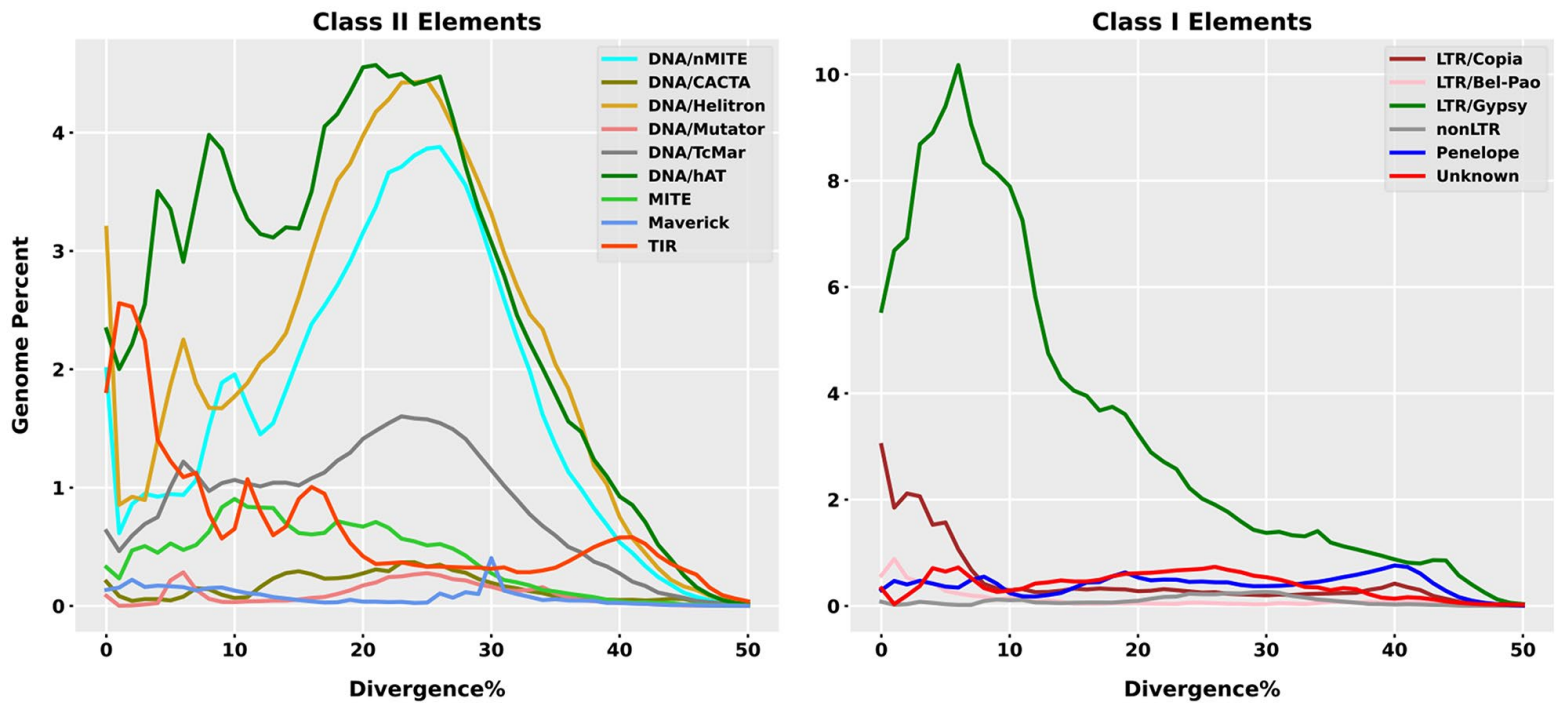
The evolutionary landscape of transposable elements (TEs) in *X. riparia*. The graph displays the proportion of the genome (%) on the y-axis and the degree of divergence based on the kimura distance on the x-axis. The K values range from 1 to 50, indicating the level of evolutionary divergence from younger to older TEs

The repeated landscape graphs were classified into Class II (DNA transposons) and Class I (retrotransposons) and revealed minor differences in their abundance and divergence within the genomes of *X. riparia* species. Class II elements (DNA transposons) that dominate the genome display a discordant repeat landscape with two or more ancient peaks for DNA/nMITE, DNA/Helitron, TIR, DNA/TcMar, and DNA/hAT families. The most abundant peaks were observed at 25% for DNA/nMITE, DNA/Helitron, TIR, and DNA/hAT. This suggests that older copies are more abundant than newly evolved copies. However, the TIR and MITE superfamilies showed the highest peaks diverging from the consensus sequence at less than 5% and 10%, respectively (Fig. 3a). Divergent peaks below 10% show accumulation and homogenization of newly emerged copies of TIR and MITE elements, and contributions to the total abundance of these elements in the genome are from recently evolved copies. Among other class II (DNA transposon) elements, DNA/CACTA and DNA/Mutator showed a flat distribution across the genome, except Maverick, which had a peak at 30% divergence. In contrast, class I elements show a more consistent repeat landscape, with peaks gradually increasing to the left of the graph, hinting at a gradual change and homogenization of newly/recently evolved copies of repeat elements. Recent peaks of LTR/Gypsy and LTR/Copia were below 10%, suggesting active dissemination and homogenization of these elements within the *X. riparia* genome. Other class I elements, including LTR/Bel-pao, nonLTR, Penelope, and unknown elements, have depicted a flat distribution across the genome without significant differences from the reference sequence (Fig. 3b). The generalized structures of each TEs superfamilies were drawn following identifications of protein domains using the DANTE tool (see Figure S2).

Age analysis of transposable elements was performed to estimate the insertion timing of major superfamilies of TEs across six chromosomes in the *Xya riparia* genome. On average, the recorded DNA transposon and retrotransposon insertions in the genome range from 25 million years ago to as recent as 5 million years ago. Recent invasion events of highly abundant class I superfamilies, including LTR/Gypsy, LTR/Copia, and LTR/Pao, were reported on six chromosomes, with insertion times ranging from 5, 0.1, and 0.2 My, respectively.

**Fig. 4:**
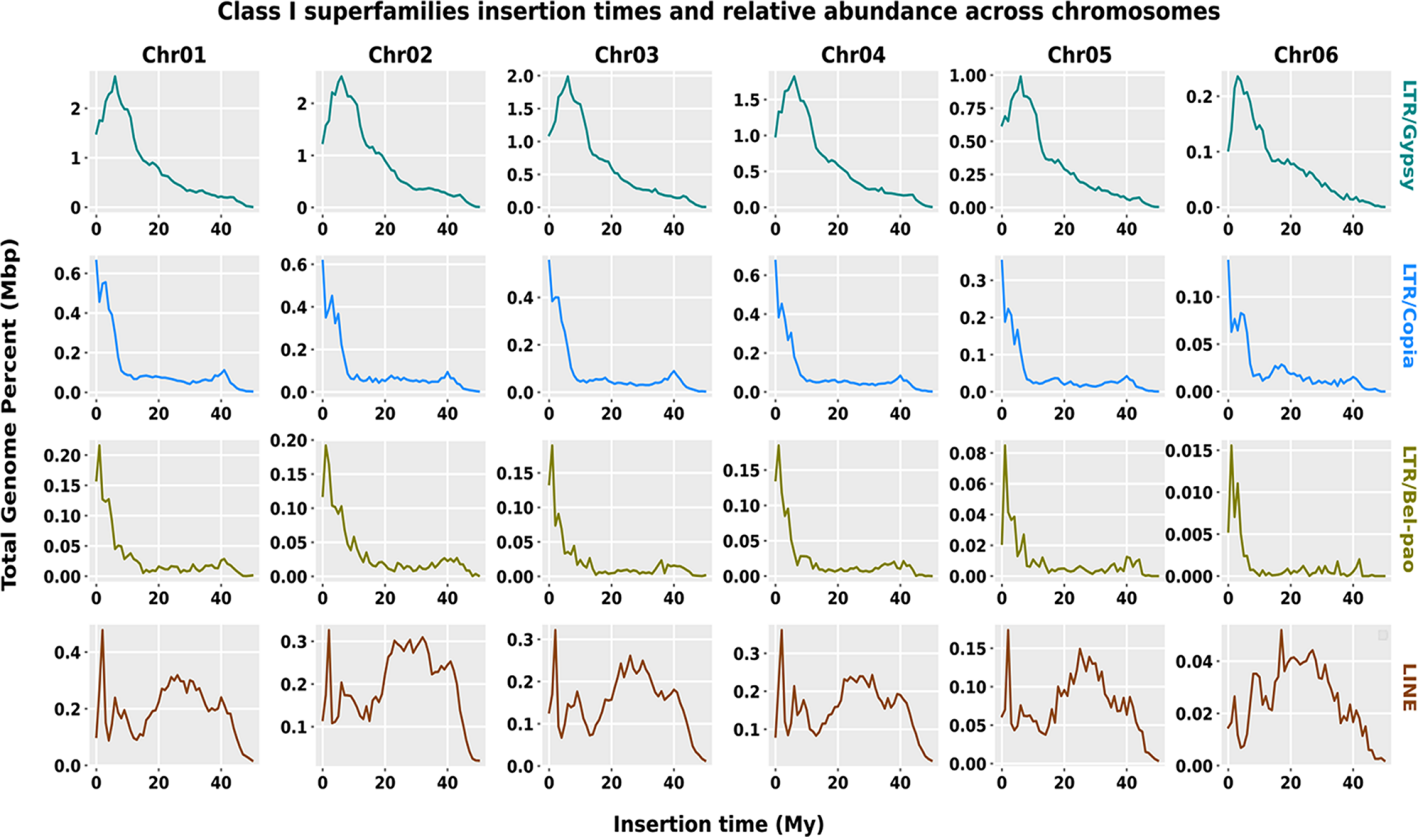
Class I superfamilies insertion times and relative abundance across the chromosomes. The TE distribution patterns are illustrated in the “1 My (million years)” bins on the x-axis. The proportion of the genome occupied by TEs is depicted on the y-axis, which is determined using the RepeatMasker align output

In contrast, the LINE superfamily showed recent and ancient invasions covering six chromosomes, with an accumulation of 1–2 My young elements to 20–30 My old elements. Recently evolved copies contributed more to the total repeat size in Chr01, Chr03, Chr04, and Chr05 than older copies. However, in Chr02, the accumulation of old and new copies demonstrated equal proportions. In contrast, Chr06 shows an explosion of LINE elements from 18 million years ago and does not show any recent accumulation. The lack of young element accumulation and the presence of highly divergent copies in Chr06 may be responsible for the smaller size (Fig. 4).

**Fig. 5:**
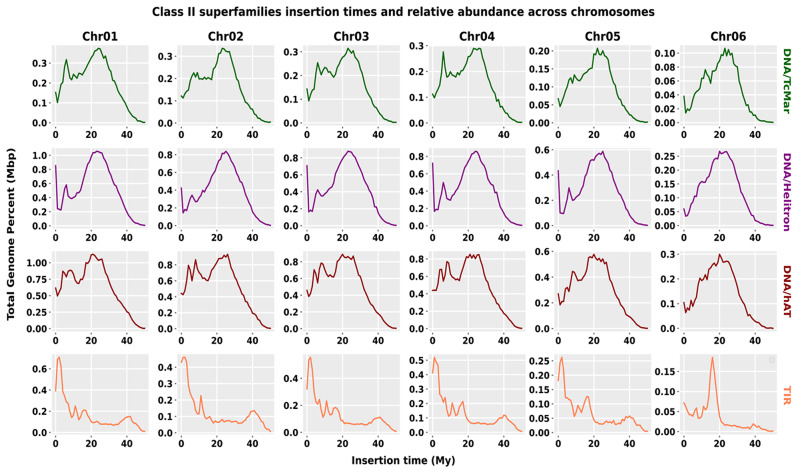
Class II superfamilies insertion times across the chromosomes. The TE distribution patterns are illustrated in the “1 My (million years)” bins on the x-axis. The proportion of the genome occupied by TEs is depicted on the y-axis, which is determined using the RepeatMasker align output

Similarly, the insertion times of four highly abundant class II superfamilies on six chromosomes have discovered the ancient burst of DNA/TcMar, DNA/Helitron, and DNA/hAT, between 24 My, 20 My, and 20 My, respectively. In contrast, the TIR superfamily showed recent invasions between 1 and 2 My across the chromosomes except for Chr06, where TIR elements burst was observed at 18 My. Older copies of the sequences mainly dominated the total size of the repeat elements in chromosomes. Minor differences in the accumulation of new and older copies were observed across the chromosomes, like, DNA/TcMar has shown not only an ancient peak but also a recent invasion at Chr04. The other families include Maverick, Penelope, MITE, DNA/nMITE, and unknown elements (Figure S3). The Maverick superfamily, in particular, exhibits an ancient invasion with an insertion time of 30 million years (My) observed on repeat graphs for all chromosomes except for Chromosome 4 (Chr04). In contrast, the Penelope superfamily exhibits both recent and ancient bursts across all six chromosomes, with older copies predominating the overall size of the superfamily. This suggests that the Penelope superfamily has persisted in the genome over long periods and has undergone repeated bursts of activity. On the other hand, the MITE superfamily shows recent bursts across the chromosomes between 8 and 10 My, except on Chr02, where it has an older burst at 21 My, and a lower accumulation rate is observed. Overall, these complex findings have important implications for understanding the dynamics and evolutionary history of TEs in the genome, providing insight into their persistence over a long time and their contribution to genome evolution (Fig. 5).

### Insights into TEs subfamilies: recent and ancient invasions

**Fig. 6:**
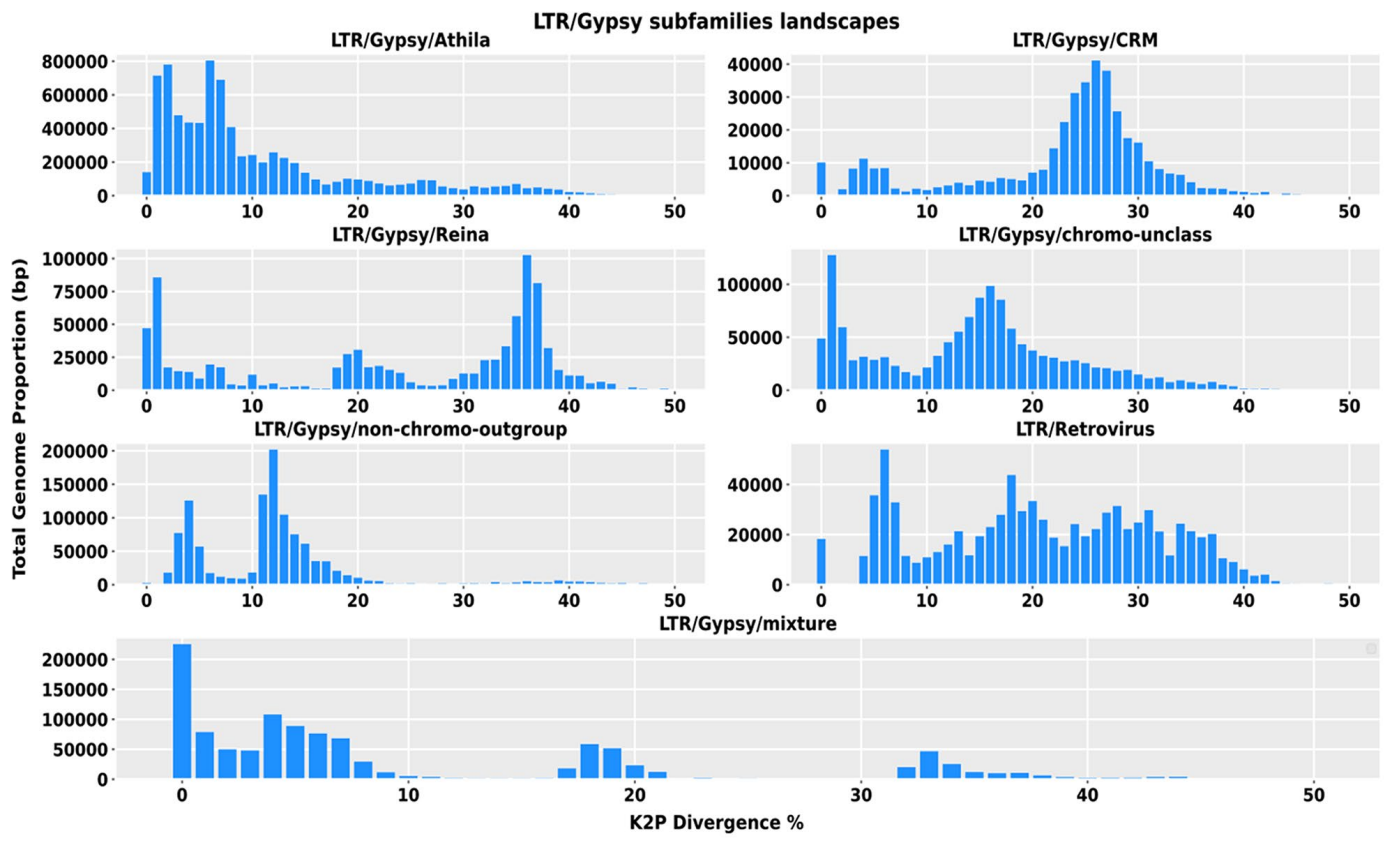
LTR/Gypsy subfamilies abundance and divergence repeat graphs. The x-axis depicts the degree of divergence, while the y-axis represents the total genome proportion. The graphs’ peaks indicate the insertion times of a particular subfamily in the genome. The graph’s right side indicates elements inserted earlier in the genome, whereas the left indicates recent insertions

**Fig. 7:**
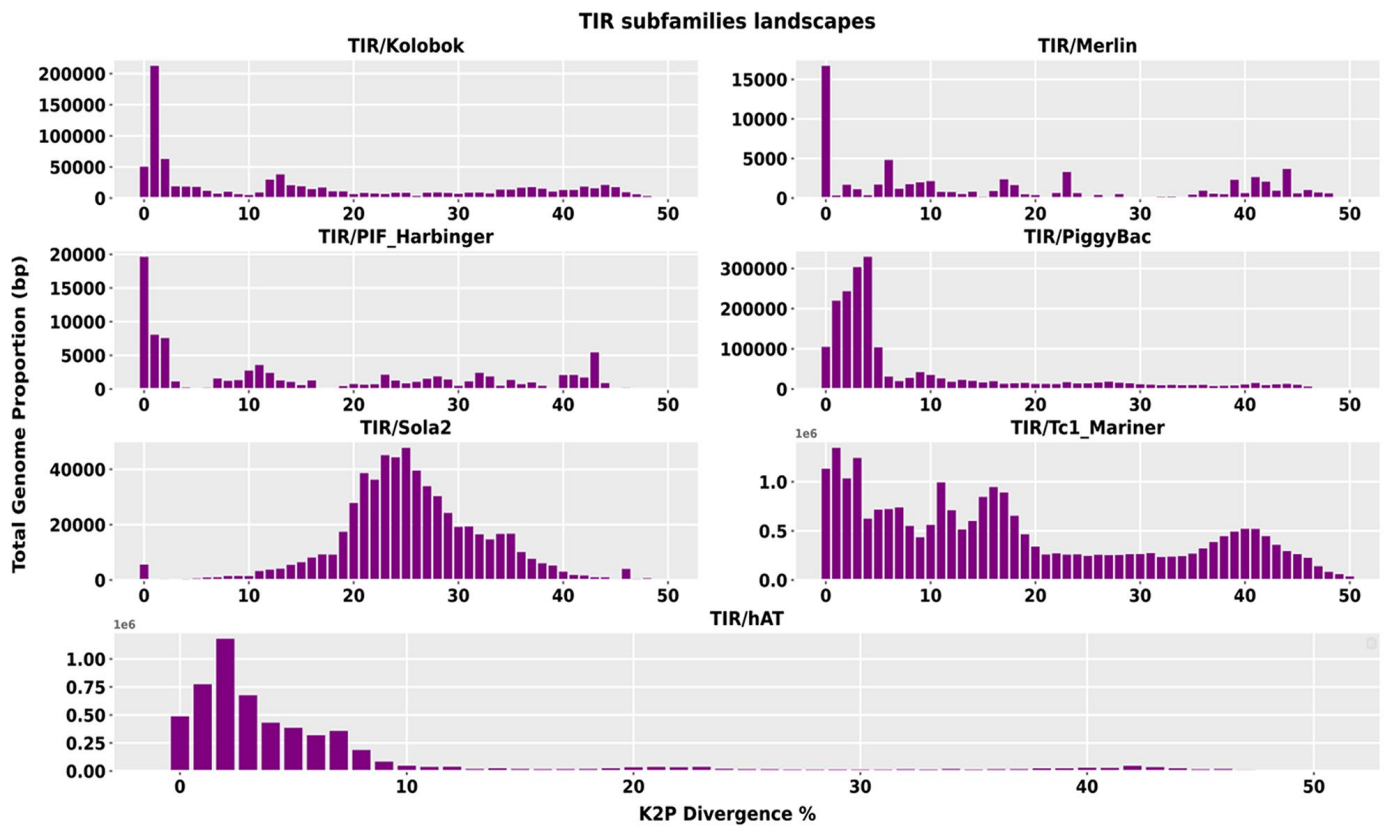
TIR subfamilies abundance and divergence repeat graphs. The x-axis depicts the degree of divergence, while the y-axis represents the total genome proportion. The graphs’ peaks indicate the insertion times of a particular subfamily in the genome. The graph’s right side indicates elements inserted earlier in the genome, whereas the left indicates recent insertions

We first subcategorized the LTR/Gypsy superfamily into seven subfamilies and assessed their divergence through repeat graphs. The LTR/Gypsy/Athila subfamily, which contains recent copies of TEs, was observed to have a single divergence peak relative to the consensus sequence at below 10%. In contrast, the LTR/Gypsy/Reina subfamily showed a double peak pattern, with a first divergence peak at 1% and an older peak at 35%. Similarly, the LTR/Gypsy/chromo and LTR/Gypsy/non-chromo subfamilies also displayed two peaks in their respective repeat graphs, with the height of the recent peak (at 1%) being more significant in the former than the latter, where the older peak was at 15%. In contrast, the subfamilies LTR/Gypsy/CRM had a single peak at 25%. Among all the LTR/Gypsy subfamilies, LTR/Gypsy/Athila was the most abundant. Specifically, LTR/Gypsy/Athila constituted nearly 50% of the total size of the LTR/Gypsy superfamily (see Fig. 6). The TIR superfamily has been subcategorized into seven subfamilies, and the divergence repeats graph of six subfamilies indicates the accumulation of recently evolved copies, except for the TIR/Sola2 subfamily, which only contains older copies. The divergence rate of TIR/Sola2 copies, relative to the consensus sequence, was between 20 and 30%, whereas the divergence rate of all other subfamilies was below 10% (see Fig. 7).

The TIR/Tc1_Mariner and TIR/hAT subfamilies have displayed a recent burst of new copies, resulting in their accumulation and high abundance in the genome. In contrast, three subfamilies, namely TIR/Kolobok, TIR/Merlin, and TIR/PIF_Harbinger, were observed to have less divergent copies, with divergence peaks at 1%, 0%, and 0%, respectively, indicating recent propagation events/bursts of these subfamilies (see Fig. 7).

The LINE superfamily has been subdivided into four subfamilies: LINE/I, LINE/R2, LINE/L1, and LINE/RTE. The divergence repeats graph of LINE/I, LINE/L1, and LINE/RTE have indicated that the older elements have accumulated towards the right side of the graph. The single divergence peaks for LINE/I, LINE/L1, and LINE/RTE have been observed at 19%, 22%, and 33%, respectively. In contrast, the subfamily LINE/R2 has demonstrated a double peak pattern, with the first peak at 14% and the second at 26%. This pattern reveals that the repeat consists of two subunits, with less divergent copies being recently active and highly divergent copies being older or inactive (Figure S4). The DNA/CACTA superfamily was also classified into two subfamilies and displayed recent and ancient invasions. The highly divergent copies of DNA/CACTA/nMITE (divergence 25%) had a larger contribution to the total size of the superfamily than the DNA/CACTA/MITE subfamily (Figure S5). Similarly, the DNA/hAT superfamily was divided into DNA/hAT/MITE and DNA/hAT/nMITE, with recent and old invasions. DNA/hAT/nMITE had a higher divergence of 20–30%, with older copies contributing more to the total size than DNA/hAT/MITE subfamily (Figure S6). The Kimura distance analysis showed that DNA/TcMar/MITE has recent copies. In contrast, DNA/TcMar/nMITE has older ones, with peak divergence rates at 10–12% and 20–30%, respectively, indicating a higher number of diverged TEs in the latter subfamily (Figure S7). Finally, the DNA/Mutator/MITE subfamily has only older copies. In contrast, the DNA/Mutator/nMITE subfamily has recent and older copies with a divergence peak of less than 10% and between 20 and 30%, respectively (Figure S8).

### Satellitome analysis

**Fig. 8:**
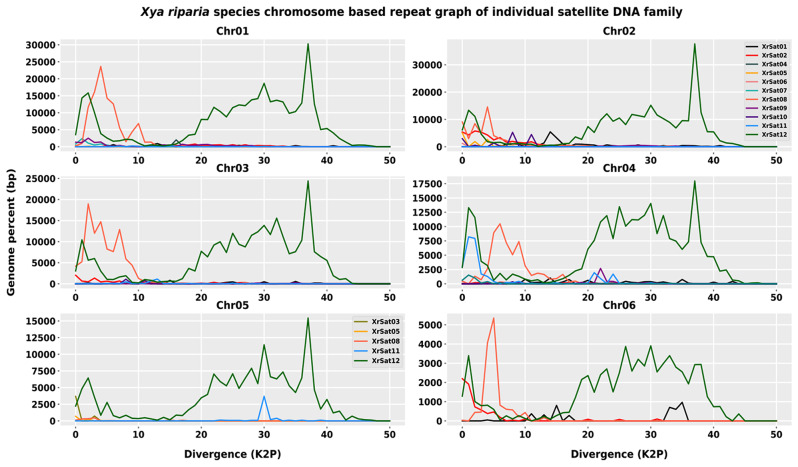
The graphs illustrate the relationship between the genome percentage and the sequence divergence of each satellite DNA family across the chromosomes. The x-axis represents the degree of divergence from the consensus sequence of the elements, while the y-axis indicates the number of copies present in the genome. Peaks on the graphs show times of insertions of a specific family in the genome. The elements inserted in the genome earlier are found on the **right** side of the graph, while recent insertions are on the **left**

In our analysis of the satellitome, we identified 14 satellite DNA families using the RepeatExplorer2 pipeline. However, the XrSat13 satellite DNA family was not recovered from the genome assembly and the unassembled raw data in the read mapping analysis of RepeatMasker and RepeatProfiler, respectively. This left us with a total of 13 satellite DNA families ranging in size from the smallest XrSat03 family (29 nt) to the largest XrSat01 family (947 nt), which is the largest satellite DNA family recorded in the Tridactalydae family so far. The A + T content of the satellite DNA families varied between 50 and 72.4%, with a median value of 59.4% (see Figure S9, Table S2). On average, the K2P genetic divergence between satellite DNA families in the genome was 8.73%, with XrSat12 being the most divergent family in the genome (with a divergence rate of 26%) and XrSat03 being the least divergent family (with a divergence rate of 0.57) (see Table S2). The graphs of the satellitome landscapes on all chromosomes showed the highest peak for the XrSat12 family above 35% K2P genetic divergence, indicating that most of the satellite DNA families have diverged from their consensus sequences. Another peak for the XrSat12 family was also observed at a divergence rate of 5%, suggesting recent activity and the homogenization of new copies. The XrSat08 family exhibited a single peak across all chromosomes except for chromosome 5, where it was absent. Some satellite families were not detected in all chromosomes but were present in some with lower abundance (see Fig. 8).

### TEs transcriptional expression analysis

**Fig. 9:**
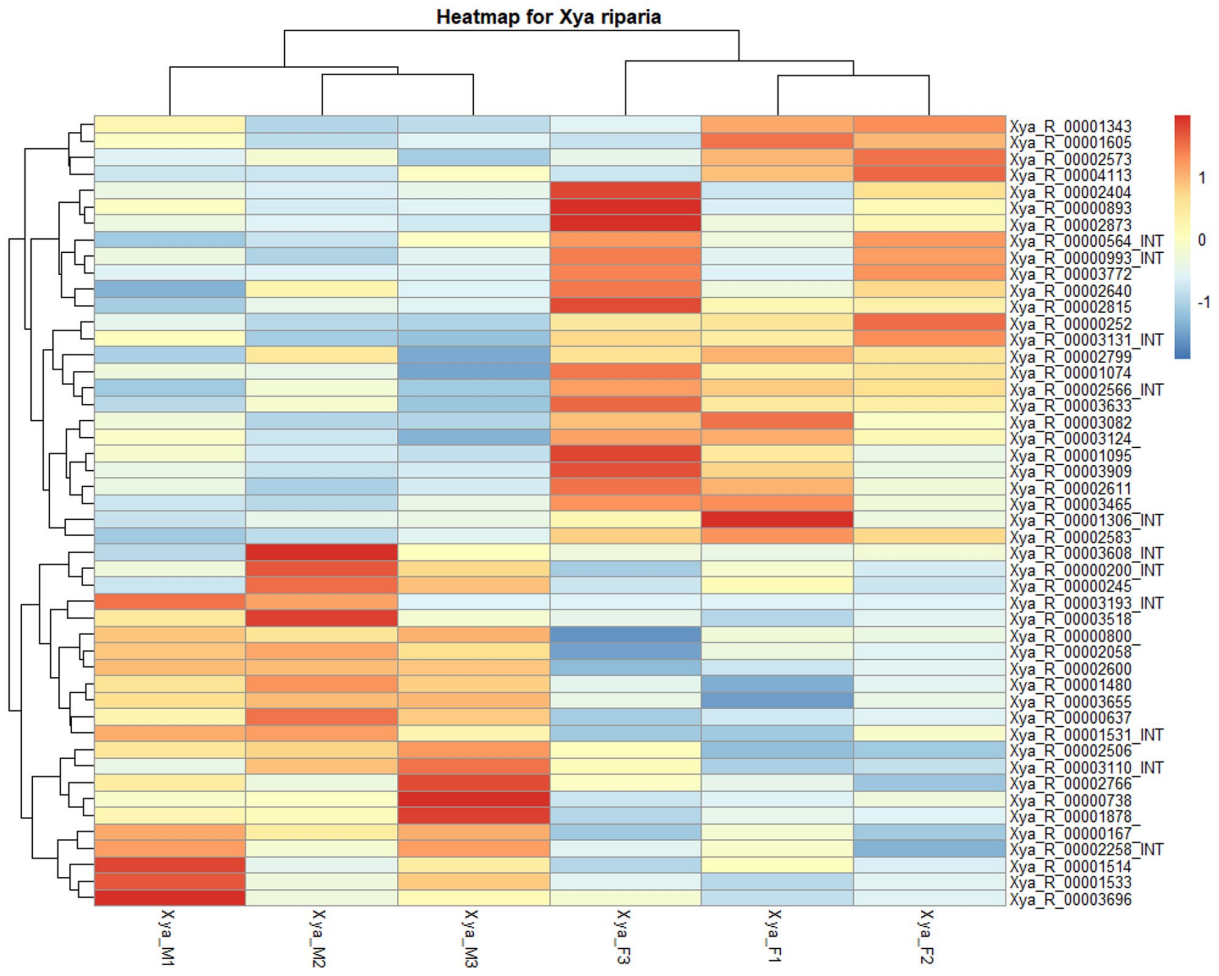
A visual representation of a heatmap illustrates the expression of 48 subfamilies of transposable elements that are differentially expressed across two samples of *X. riparia*

In the analysis of TEs (transposable elements) expression, we observed that 2662 and 2548 TEs were expressed with a non-zero total read count in female (ovary) and male (testes) reproductive tissue samples of *X. riparia*, respectively. The proportion of TE expressed in the two samples was biased toward males, as a higher number of TEs were expressed in males than females. We have observed 3228 expressed TEs with the non-zero total read count in all reproductive samples of *X. riparia* male and female. We use the recommended differential expression parameters of the p-value (*p* = 0.05) and log2FoldChange (log2fc = + 1, -1) to determine the differentially expressed TEs subfamily in all samples. We found that 48 out of the 3228 TEs sequences belongs to different superfamilies were differentially expressed in all samples, up or down. The heatmap shows the expression data of 48 differentially expressed TEs across all samples from males and females of *X. riparia* species. More Class II elements exhibited differential expression (upregulated or downregulated) than Class I elements. The hAT/nMITE elements were the most prominent among DNA transposons, while the LTR/Gypsy elements were the most differentially expressed TEs among retroelements. However, no differentially expressed satellite DNA family was found (see Fig. 9). The details of 48 differentially expressed TEs, including their p-values, log2fold changes, and corresponding families, are provided in the supplementary information (see Table S3).

Similarly, the boxplots for all TE clades indicate that certain transposable elements are differentially expressed. Outliers representing these differentially expressed TEs can be seen outside the whiskers in some clades, including LTR, LINE, Penelope, TIR, Helitron, and DNA transposon. A total of 48 TEs showed differential expression in the two RNA-seq samples, each with three biological replicates (see Figure S10, S11).

## Discussion

Pygmy mole crickets (family: Tridactylidae) are not considered true crickets. Although they belong to Orthoptera, they are placed in a different suborder than true crickets. Pygmy mole crickets are part of the suborder Caelifera, which includes grasshoppers and locusts, while true crickets belong to the suborder Ensifera.

### TEs abundance and comparison with related species

The study analyzed the genome of *Xya riparia* species to determine its genome size and the abundance, distribution, and age of its transposable elements (TEs). The genome size was 1.67 GB, and the repeatome analysis identified 731.63 Mb (44%) of repetitive sequences. Notably, the percentage of repetitive DNA sequences found in pygmy mole cricket species, including *X. riparia*, was found to be similar to that observed in the ensifera suborder species *Teleogryllus occipitalis*, where estimated repeats accounted for 44.75% [29]. However, within the caelifera suborder, *X. riparia* exhibited higher repeat sequences compared to species such as *Gryllus bimaculatus* and *Laupala kohalensis*, which reportedly accounted for 28.9% and 34.5% repetitive sequences, respectively [30]. Contrastingly, species such as *Locusta migratoria* and *Schistcerca gregaria* demonstrated a higher percentage of repetitive sequences in their genome assemblies, with reported percentages of 60% and 62%, respectively [31]. Similarly, morabine grasshoppers displayed a large diversity of TEs and satellite DNA, constituting between 66 and 75% of the genome assembly, surpassing the proportion reported in *X. riparia* [28]. Likewise, the repetitive DNA reported in the genome of *Angaracris rhodopa* accounted for 74.56% of the whole genome [11]. These findings underscore the variability in repetitive DNA content among different insect species, reflecting diverse evolutionary trajectories and selective pressures within various lineages.

### Dominance of DNA transposons in *Xya riparia* and insects

Our analysis revealed that a significant portion of the *X. riparia* genome comprised DNA transposons. Similarly, some other Insects exhibit a predominant presence of DNA transposons over retrotransposons, owing to the high proportion of DNA transposons within their genomes coupled with their active transposition, while retrotransposons are typically found in low numbers and are frequently silenced. This characteristic disparity in transposable element (TE) composition within insect genomes underscores the dynamic nature of TE dynamics and their potential impact on genomic evolution [32, 33]. These findings align consistently with prior studies conducted on the genomes of other insect species, such as house crickets (*Acheta domestica*) and gomphocerine grasshoppers, where DNA transposons were identified as the predominant repetitive elements [9, 34]. Furthermore, in Hymenoptera, another significant group of insects, DNA transposons were similarly found to be more prevalent. Studies on species such as *Harpegnathos saltator* and *Pediculus humanus* (human body louse) reported contributions of up to 35.25% in the former and 44.43% in the latter, further emphasizing the prevalence of DNA transposons as major constituents of the repetitive element landscape in insects [26].

### Abundance and diversity of retrotransposon superfamilies

Among retrotransposons, the superfamilies LTR/Gypsy and LTR/Copia were highly abundant, with LTR/Gypsy being the most predominant superfamily in the genome. We identified the highest number of full-length copies of LTR/Gypsy, LTR/Copia, and LTR/Bel-pao, respectively. This observation suggests that retrotransposons possess all the essential protein domains required for their transposition within the genome, which likely contributes to their high abundance. Moreover, the presence of full-length and less divergent copies of LTR/Gypsy indicates its active involvement within the genome (see Fig S12, Table S4). In contrast, we did not observe any full-length copies of LINE elements; instead, we found fragmental amplifications and highly divergent copies within the genome. This could be the reason LINE elements were less abundant compared to other superfamilies. However, retrotransposons, especially LINE elements, were found to dominate in some species, such as *Schistocerca gregaria*, where LINE elements accounted for 27.6% of the genome, and in *A. rhodopa*, where LINE elements made the highest contribution. [11, 31]. Additionally, some more studies have reported contradictory results to our findings, such as in the transposable elements study of 26 Drosophila species, where LTR and LINE elements were observed to proliferate in most species [35]. The reasons why a particular type of repetitive DNA may proliferate in genomes are somewhat understood, including factors such as transposition mechanisms, target site preferences, and natural selection. However, further research is needed to expand our knowledge on this topic [36–39].

### Chromosome-level variation and age analysis of TEs

TEs can significantly impact the structure and function of chromosomes, and we observed a positive correlation between chromosome size and TE content. Chromosome-level differences in TE content have been observed across various species and can contribute to phenotypic variation and speciation and even be involved in the sex chromosome determination of the species [40, 41]. In the current work, the repeatome analysis also revealed interesting patterns in the distribution and abundance of transposable elements across the six chromosomes of the *Xya riparia* genome. The study found a high percentage of LTR/Gypsy, DNA/nMITE, DNA/hAT, DNA/Helitron, LINE, and DNA/TcMar elements on the six chromosomes, with LTR/Gypsy being the most abundant on chromosome 2 (Fig. 2). A similar study has been reported, which states that the accumulation of transposable elements at the chromosome level is responsible for the differences in size observed among the polymorphic Y chromosomes in Drosophila [42]. A study comparable to our research indicated that a marked rise in non-coding repetitive sequences and transposable elements (TEs) was evident in most W/Y chromosomes [43, 44].

We conducted an age analysis of transposable elements (TEs), which provided insights into the invasion times of four highly abundant retrotransposon and DNA transposons superfamilies across six chromosomes, ranging from 0.25 to 5 million years (My) (Figs. 3 and 4). This observed pattern suggests that the expansion of transposable elements (TEs) in the *X. riparia* genome primarily results from ongoing TE activity. The LINE insertion time observed on chromosome 6 is older compared to other chromosomes, but the reasons for this difference remain unclear here. Differential rates of genomic turnover or repair mechanisms operating on different chromosomes may result in variations in the preservation and accumulation of transposable elements (TEs) over time [45–47]. Furthermore, a similar analysis of TE age distributions in Trichoptera species showed that LINEs, DNA transposons, and LTRs have a sequence divergence of 0–10% within copies of a particular repeat [48]. In contrast, the analysis of TE age in *Spodoptera frugiperda* demonstrated that PIF/Harbinger has some older/degenerated copies that are around 30–35 million years old [49].

### Satellitome analysis

The analysis of the satellitome is important for understanding the genomic organization and evolution of insects and has important implications for fields such as sex chromosome biology. The present study identified 14 satellite DNA families in the genome of *X. riparia*. The number and proportion of satellite DNA in insects vary, with the genus Calliptamus having 20 satellite DNA families [50]. Other insects have reported a higher number of satellite DNA families than *X. riparia*, such as 62 in *L. migratoria* [51], 76 in *Pyrgomorpha conica* grasshoppers [52], 45 in *Eneoptera surinamensis* [53], 29 in Ladybird Beetle [54], 53 in *Ronderosia bergii* [55], 188 in Drosophila species [56], and 92 in morabine grasshopper [28]. In contrast, some insect species have a lower number of satellite DNA families, such as 12, 9, and 9 in *Rhammatocerus brasiliensis*, *Schistocerca rubiginosa*, and *Tribolium castaneum*, respectively [57, 58]. Satellite DNA varies in insects due to a number of factors, including genetic drift, selection, and chromosomal rearrangements. Overall, the variation in satellite DNA observed in insects reflects the complex interplay between genetic, environmental, and evolutionary factors that shape genome structure and organization over time.

## Conclusion

In conclusion, our study offers significant insights into the genomic characteristics of *X. riparia*, highlighting its highly complex nature primarily due to the abundance of transposable elements (TEs). With a genome size of 1.67 Gb, approximately 731.63 Mb of which comprises repetitive sequences, the presence of TEs underscores the dynamic evolutionary processes shaping this species genome. The genome was dominated by DNA transposons compared to retrotransposons, with highly divergent TE copies as compared to less divergent copies of retrotransposons. The high abundance of DNA transposons could be a result of sufficient time for sequence accumulation. TEs were almost evenly distributed across the six chromosomes, with some exceptions showing variations in abundance and insertion times. We observed active retrotransposons within the genome, as reflected in the divergence profiles and confirmed through the identification of full-length copies containing all necessary protein domains for retro-transposition. Similarly, retrotransposons exhibited differential expression in male and female tissues. We did not observe any significant variations in satellite DNA repeats within the genome in terms of distribution and abundance across the chromosomes and the entire genome. Overall, our study demonstrates the intricate interplay between transposable elements and the genomic landscape of *X. riparia*, shedding light on the evolutionary dynamics and significance of TEs elements. Together, these findings contribute to a deeper understanding of the genomic biology of *X. riparia* and lay the foundation for future research exploring the role of transposable elements in insect genome evolution and functionality. **Methods**.

### Preparation of data through quality check, random sampling, and pre-processing

We used genome survey data to compare satellite DNA repeat analysis between male and female Xya riparia genomes and utilized the RepeatExplorer2 pipeline. To ensure representative sampling of the entire genome, we randomly sampled 0.5x genome coverage using the SeqTK tool (https://github.com/lh3/seqtk, accessed online, 01 January 2023) with a recommended genome coverage range of 0.01-0.5x. We extracted 5 million reads from each sample, which were then uploaded to RepeatExplorer2 galaxy. The data quality was assessed using the FastQC tool within the RepeatExplorer2 Galaxy instance, and the pre-processing of fastq files was conducted using the “preprocessing of fastq paired reads” tool with default settings. This pre-processing included trimming, quality filtering, discarding single reads, retaining complete pairs, cut-adapt filtering, and interlacing two fastq files. To facilitate downstream analysis, we added species-specific four-letter prefixes to the read names using the ‘RepeatExplorer Utilities → FASTA read name affixer’ tool, running it on each output file. Subsequently, we employed the ‘RepeatExplorer Utilities → Read sampling’ tool to randomly sample 1,000,000 paired reads from the interlaced files. Finally, the ‘Text manipulation → Concatenate datasets’ tool was used to create a FASTA file containing 2 million reads, sequentially selecting and inserting files with coded reads from the two samples (XyaM and XyaF). This resulting file served as the input for subsequent analysis steps.

### RepeatExplorer2 and TAREAN clustering analysis

We utilized the previously generated concatenated FASTA file as the input data for clustering analysis using the RepeatExplorer2 and TAREAN (tandem repeat analyzer) tools, which can be accessed at http://repeatexplorer.org/?page_id=818. For the comparative mode of RepeatExplorer2 clustering, we configured the parameters as follows: pair-end reads set to yes, sample size set to 2 million reads, reference database set to Metazoa version 3.0, select queue set to “long,” and enabled the comparative analysis option in the advance options, with a group code length of ‘4’. Both clustering analyses produced three files: a log file, an HTML report, and an HTML archive report. To further examine the results, we downloaded the HTML archive reports.

### Annotation of transposable elements using the FasTE pipeline

TE annotation of *Xya riparia* was performed using the FasTE pipeline, designed to be a quick guide for de novo transposable element (TE) library generation and subsequent TE screening [59]. First, we have generated a TE library utilizing the package; Extensive de novo TE Annotator (EDTA), which combines ab initio and homology-based methods to identify TEs [60]. The TE library generated in the previous step was analyzed using DeepTE, a method that employs a convolutional neural network to classify transposons with unknown classification [61]. Although DeepTE improved the annotation of unknown elements, there were still a considerable number of unknown sequences. To further classify the unknown elements, we utilized TEsorter [62], a tool designed to classify unknown transposable elements, including both Class I and Class II elements. REXdb was used as a reference database to classify these unknown elements, and this significantly enhanced the annotation of these elements. This refined TE library was then used for subsequent downstream analysis.

To perform TE screening on the *Xya riparia* genome, we used the RepeatMasker software and the custom repeat library option. RepeatMasker was run on the whole genome and each chromosome with specific parameters, including -s (slow), -a (alignment file), -gff (gff file), -e (search engine) rmblast, -pa 24, and -lib (custom repeat library). The screening allowed us to search for abundance and divergence differences across the chromosomes and perform divergence and abundance analysis for satellite DNA repeats. To determine the insertion time of each element across the chromosomes, age analysis was performed using the script provided for RepeatMasker result parsing. This comprehensive approach provided an accurate and detailed analysis of the mobilome of *Xya riparia* at the chromosome level.

### TEs protein domains extraction and satellite DNA repeat profiling

We used the RepeatExplorer Galaxy Platform to extract consensus sequences of transposable elements using the DANTE tool. The DANTE tool was executed with the metazoan database, BLOSUM80 scoring matrix, and zero iterative searches. As a result, three output files were generated in filtered and full gff3 and fasta formats. Subsequently, the filtered gff3 file was processed using the “Summarize gff3 output from DANTE” tool to obtain a summary of protein domains and their respective frequencies for each transposable element family. The “Extract Domains Nucleotide Sequences” tool was employed to obtain the consensus sequences of the transposable elements. The resulting sequences were compiled into a final file, renamed, and used as the input FASTA file for subsequent analysis using the Repeat Profiler program. The RepeatProfiler tool was used to create, visualize, and compare repetitive DNA profiles of each satellite DNA from low-coverage short-read sequence data of male and female species of *X. riparia*. To utilize Repeat-Profiler, two inputs were necessary. The first input was genome survey data in the form of FASTQ files containing short-read sequence data from each sample, for which we used 2x data for this analysis. The second input was FASTA files containing reference sequences for the repeats that needed to be evaluated. The reference sequences in satellite DNA fasta files were mapped against 5 million randomly selected reads from each sample to analyze and compare satellite DNA profiles between the two samples. The “pre-corr” flag was used for correlation analysis to generate the required input file (user_groups.txt). All other settings were maintained as default throughout the analysis.

### Transcriptional expression analysis

We used Illumina RNA-seq reads from males and females of *Xya riparia* species to investigate the transcriptional profile of TEs in each tissue. *Xya riparia* RNA-seq data of females and males was already uploaded with NCBI/SRA with BioProject number PRJNA763707. We have quantified TEs expression in the Xya riparia male and female samples through RNA_seq reads mapping to Transposable elements (TEs) copies using SalmonTE (https://github.com/hyunhwaj/SalmonTE, accessed online, 25, January 2023) and DESeq2. TEs used as a reference for quantification from RNA_seq data were obtained by combining the newly characterized TEs from the current experiment and orthoptera order TEs from the Repbase. Differential expressions, boxplots, and heatmaps were done using DESeq2.

### Electronic supplementary material

Below is the link to the electronic supplementary material.

Supplementary Material 1

## Data Availability

We deposited the data and materials supporting the findings of this study in the National Center for Biotechnology Information (NCBI) repository, under BioProject accession number PRJNA763707.
