# Supplementary material for: Comprehensive analysis of the Xya riparia genome uncovers the dominance of DNA transposons, LTR/Gypsy elements, and their evolutionary dynamics
Source: BMC Genomics. 2024 Jul 12;25:687. doi: 10.1186/s12864-024-10596-5 (PMC11245825; doi:10.1186/s12864-024-10596-5)
Supplement: Supplementary file 1 — Supplementary Material 1 [file 12864_2024_10596_MOESM1_ESM.docx]

**Additional Information**

**Comprehensive analysis of the *Xya riparia* genome uncovers the dominance of DNA transposons and LTR/Gypsy elements and their evolutionary dynamics**


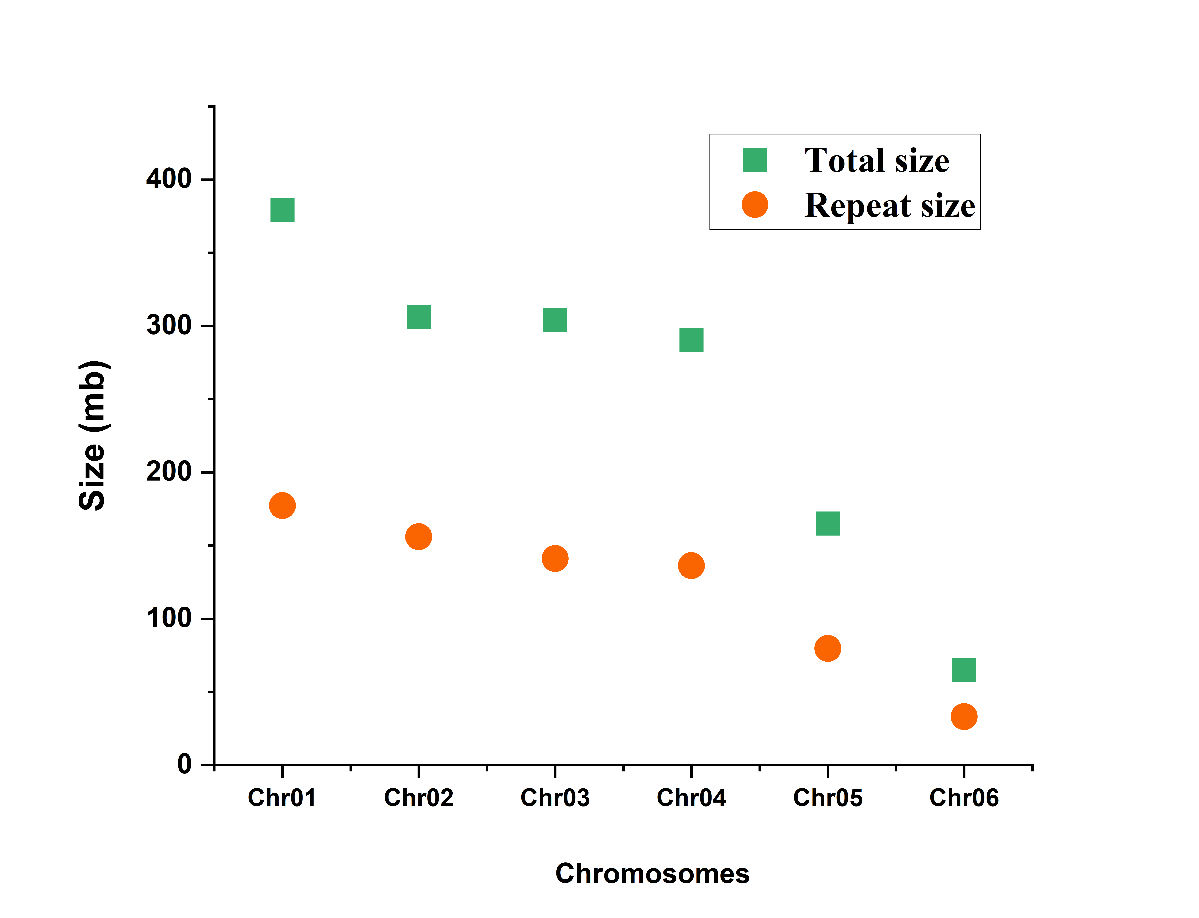


**Figure S1: A scatter plot depicts the relationship between total chromosome size and repeat size.** X-axis of the graph represents the name of the chromosomes and Y-axis represents the total genome size of each chromosome.


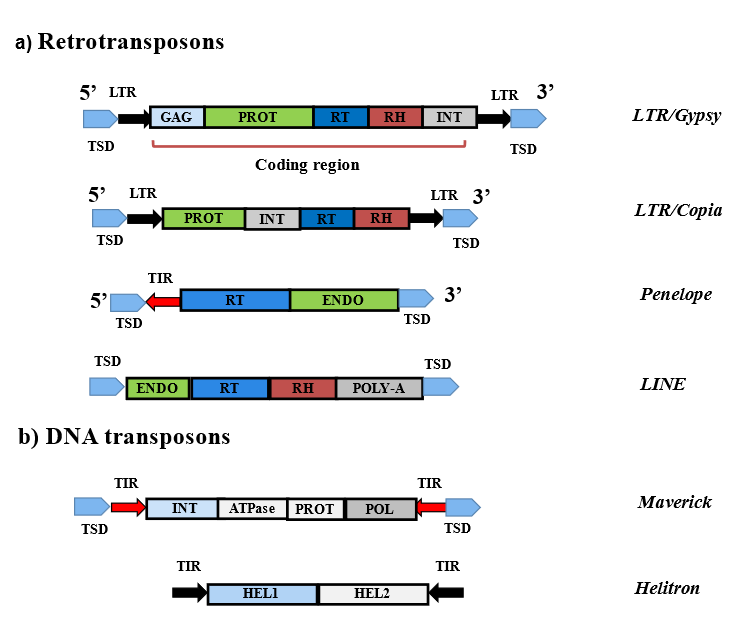


**Figure S2: The graph displays a generalized structure of the TE families within the *X. riparia* genome.** Protein domains present in each family were identified and extracted using the DANTE tool.

The study of the insertion times of other transposable elements (TE) superfamilies has yielded intriguing results for six chromosomes. These include the Maverick, Penelope, MITE, DNA/nMITE, and unknown elements. Notably, the Maverick superfamily is characterized by an ancient invasion with an insertion time of 30 million years (my) on repeat graphs for all chromosomes, except for Chromosome 4 (Chr04). Here, a double peak pattern is evident, with recent 1 My and older invasions at 30 My. This pattern suggests that the superfamily may have undergone different evolutionary trajectories on Chr04 than on other chromosomes.

In contrast, the Penelope superfamily exhibits both recent and ancient bursts across all six chromosomes, with older copies predominating the overall size of the superfamily. This suggests that the Penelope superfamily has persisted in the genome over long periods of time and has undergone repeated bursts of activity. The MITE superfamily, on the other hand, shows recent bursts across the chromosomes between 8-10 my, except on Chr02, where it has an older burst at 21 my, and a lower rate of accumulation is observed. These findings suggest that the MITE superfamily has undergone different patterns of activity and accumulation across different chromosomes. Overall, these complex findings have important implications for understanding the dynamics and evolutionary history of TEs in the genome, providing insight into the distinct properties of different TE superfamilies and their contribution to genome evolution.

**

Figure S3: Others Class I and Class II superfamilies insertion times across the chromosomes.** The TE distribution patterns are illustrated in bins of “1 My (million years)” on the x-axis. The proportion of the genome that is occupied by TEs is depicted on the y-axis, which is determined using the RepeatMasker align output.

LINE superfamily was subclassified into four subfamilies, LINE/I, LINE/R2, LINE/L1, and LINE/RTE. LINE/I, LINE/L1, and LINE/RTE have shown accumulation towards the right side of the graph which indicates the presence of older elements. The single divergence peaks for LINE/I, LINE/L1, and LINE/RTE were observed at 19%, 22%, and 33%, respectively, accumulation of a number of copies was observed after the divergence peaks for subfamilies LINE/I and LINE/L1 but for subfamily LINE/RTE copies were observed before the divergence peak. In contrast, the subfamily LINE/R2 has shown a double peak pattern with the first peak at 14% and the second peak at 26%. This pattern indicates the presence of two subunits of a repeat less divergent copies that are recently active and highly divergent are older/inactive. The overall size of the LINE superfamily was highly dependent on LINE/L1 as it presents a double amount in the genome as compared to LINE/I and LINE/R2.


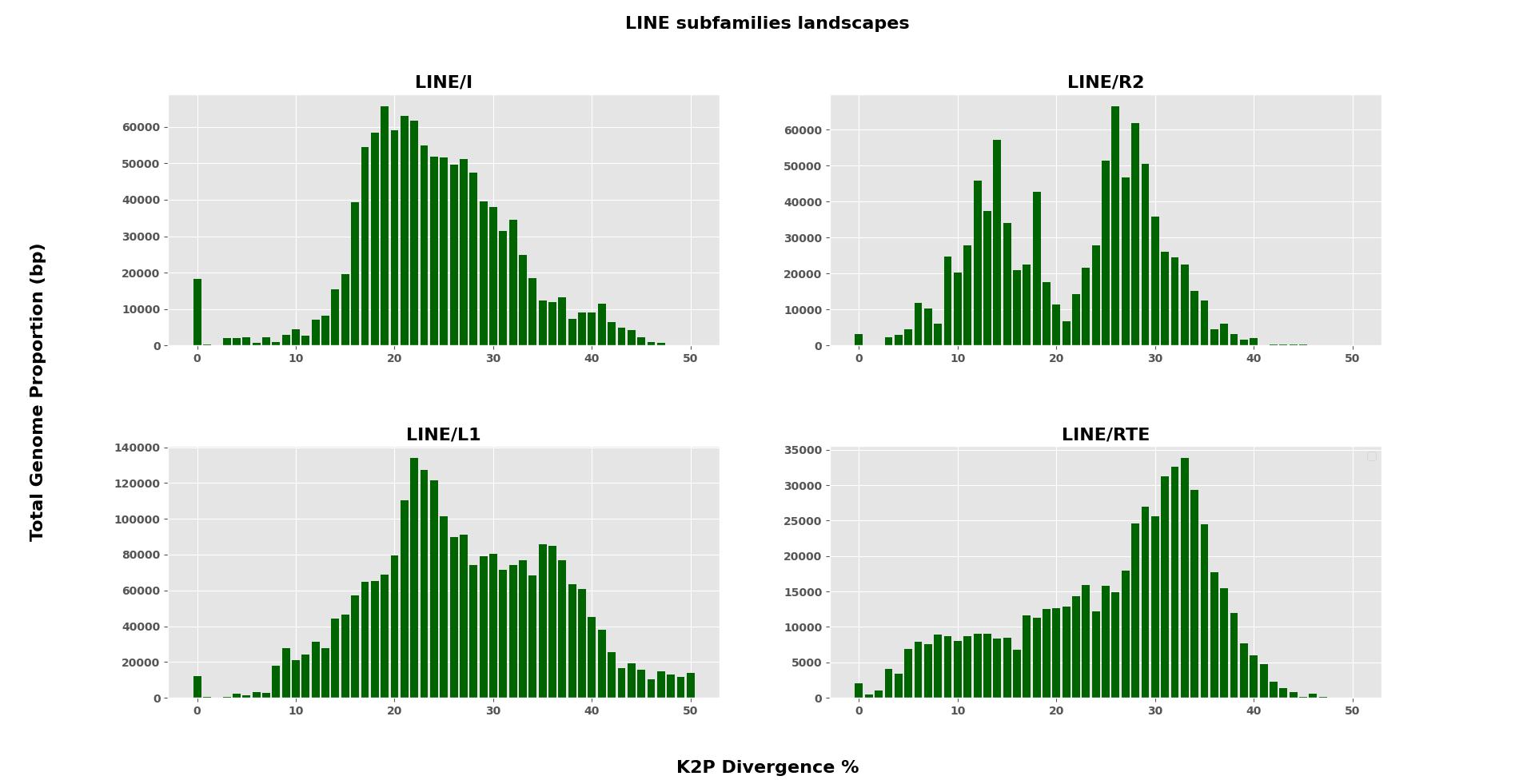


**Figure S4: LINE subfamilies abundance and divergence repeat graphs.** The x-axis depicts the degree of divergence, while the y-axis represents the total genome proportion. The peaks on the graphs indicate the times of insertion of a particular subfamily in the genome. The right side of the graph indicates elements that were inserted earlier in the genome, whereas the left side indicates recent insertions.

We have subcategorized superfamilies and assessed their prevalence and divergence to analyze their distribution throughout the genome and their overall contribution to the size of the superfamilies. The classification of DNA transposon superfamilies was based on the presence of MITE or nMITE sequences. The DNA/CACTA superfamily was subdivided into DNA/CACTA/MITE and DNA/CACTA/nMITE, and the repeat graph showed recent and ancient invasions, respectively. The copies of DNA/CACTA/nMITE elements were highly divergent, with a divergence peak at 25%, and had a greater contribution to the total size of the superfamily compared to the DNA/CACTA/MITE subfamily, which contained fewer copies but showed an accumulation of recently evolved copies.


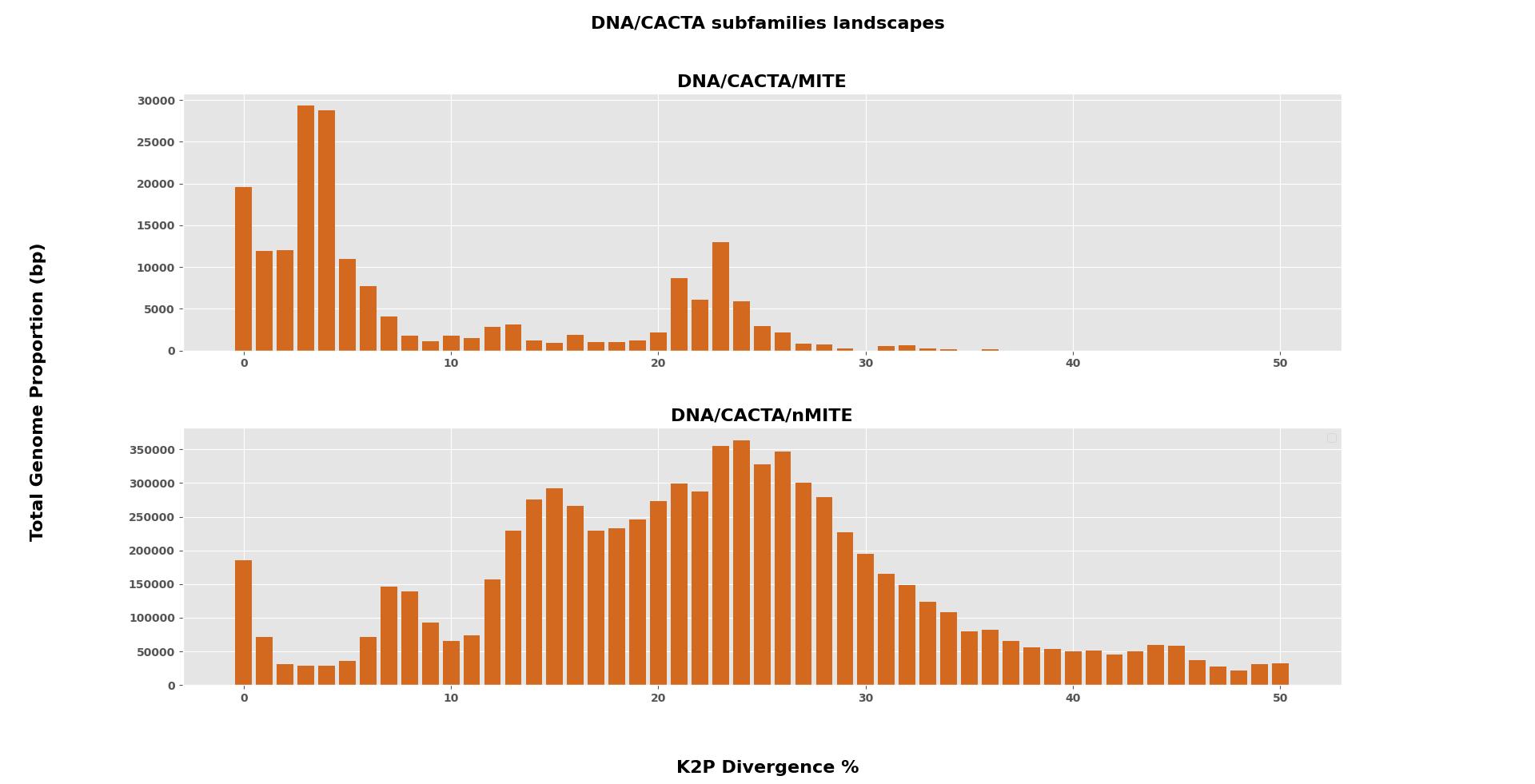


**Figure S5: DNA/CACTA subfamilies abundance and divergence repeat graphs.** The x-axis depicts the degree of divergence, while the y-axis represents the total genome proportion. The peaks on the graphs indicate the times of insertion of a particular subfamily in the genome. The right side of the graph indicates elements that were inserted earlier in the genome, whereas the left side indicates recent insertions.

The DNA/hAT superfamily was subclassified into DNA/hAT/MITE and DNA/hAT/nMITE. We found that each subfamily reflected both recent and old invasions on the repeat graph. Additionally, we found that the divergence of DNA/hAT/nMITE elements from the consensus sequence ranged between 20% to 30%, which is an indication of the presence of older copies within the genome. These older copies made a greater contribution to the overall size of the DNA/hAT superfamily compared to the DNA/hAT/MITE subfamily, which was found to contain a greater number of recently evolved copies.


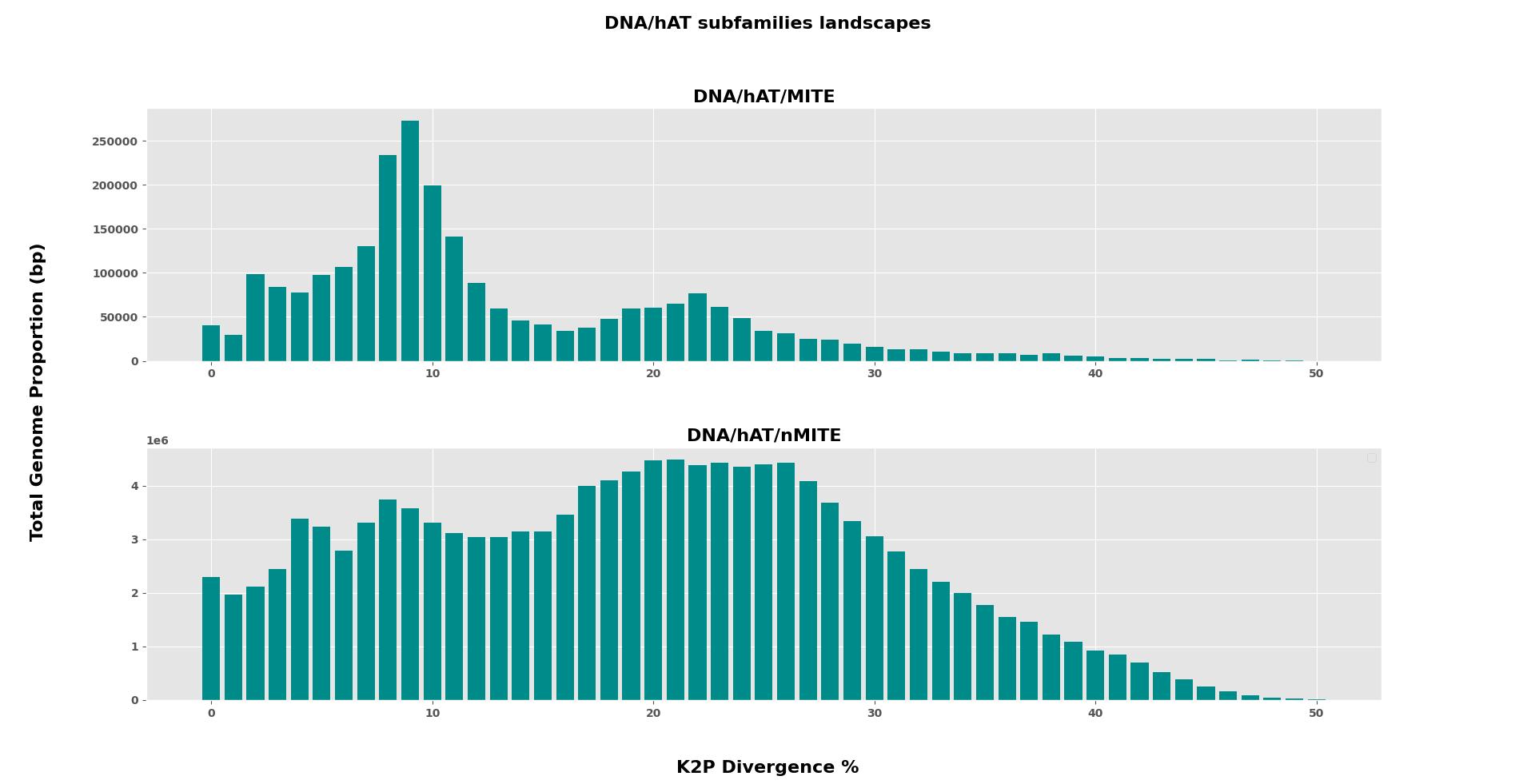


**Figure S6: DNA/hAT subfamilies abundance and divergence repeat graphs.** The x-axis depicts the degree of divergence, while the y-axis represents the total genome proportion. The peaks on the graphs indicate the times of insertion of a particular subfamily in the genome. The right side of the graph indicates elements that were inserted earlier in the genome, whereas the left side indicates recent insertions.

The Kimura distance landscape analysis has revealed that the subfamily DNA/TcMar/MITE contains comparatively recent copies, whereas the DNA/TcMar/nMITE copies present in the genomes are relatively older. The sequence divergence in relation to the consensus sequences is observed to peak between 20-30% for the DNA/TcMar/nMITE subfamily, whereas for the DNA/TcMar/MITE subfamily, the peak divergence rate is around 10-12%. This provides a clear indication that the DNA/TcMar/nMITE subfamily has a higher number of diverged copies of transposable elements (TEs).


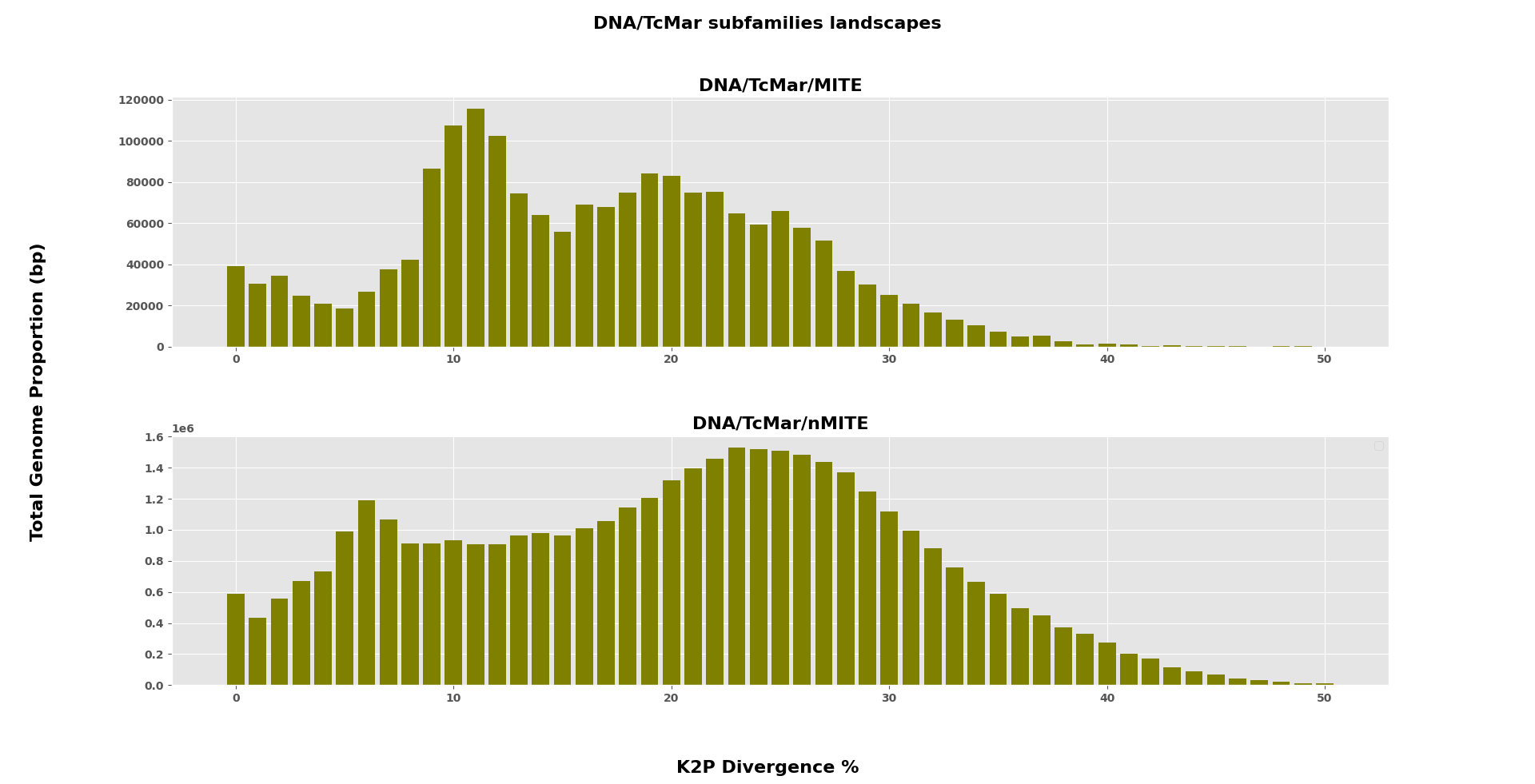


**Figure S7: DNA/TcMar subfamilies abundance and divergence repeat graphs.** The x-axis depicts the degree of divergence, while the y-axis represents the total genome proportion. The peaks on the graphs indicate the times of insertion of a particular subfamily in the genome. The right side of the graph indicates elements that were inserted earlier in the genome, whereas the left side indicates recent insertions.

The DNA/Mutator/MITE subfamily has ancient invasions, whereas the DNA/Mutator/nMITE subfamily has recent invasions (divergence peaks less than 10%) as well as the presence of earlier copies (divergence peaks between 20-30%) in the genome. This clearly demonstrates that the DNA/Mutator/nMITE subfamily contains a greater number of both recently active and older divergent copies; however, the other subfamily only contains older copies.


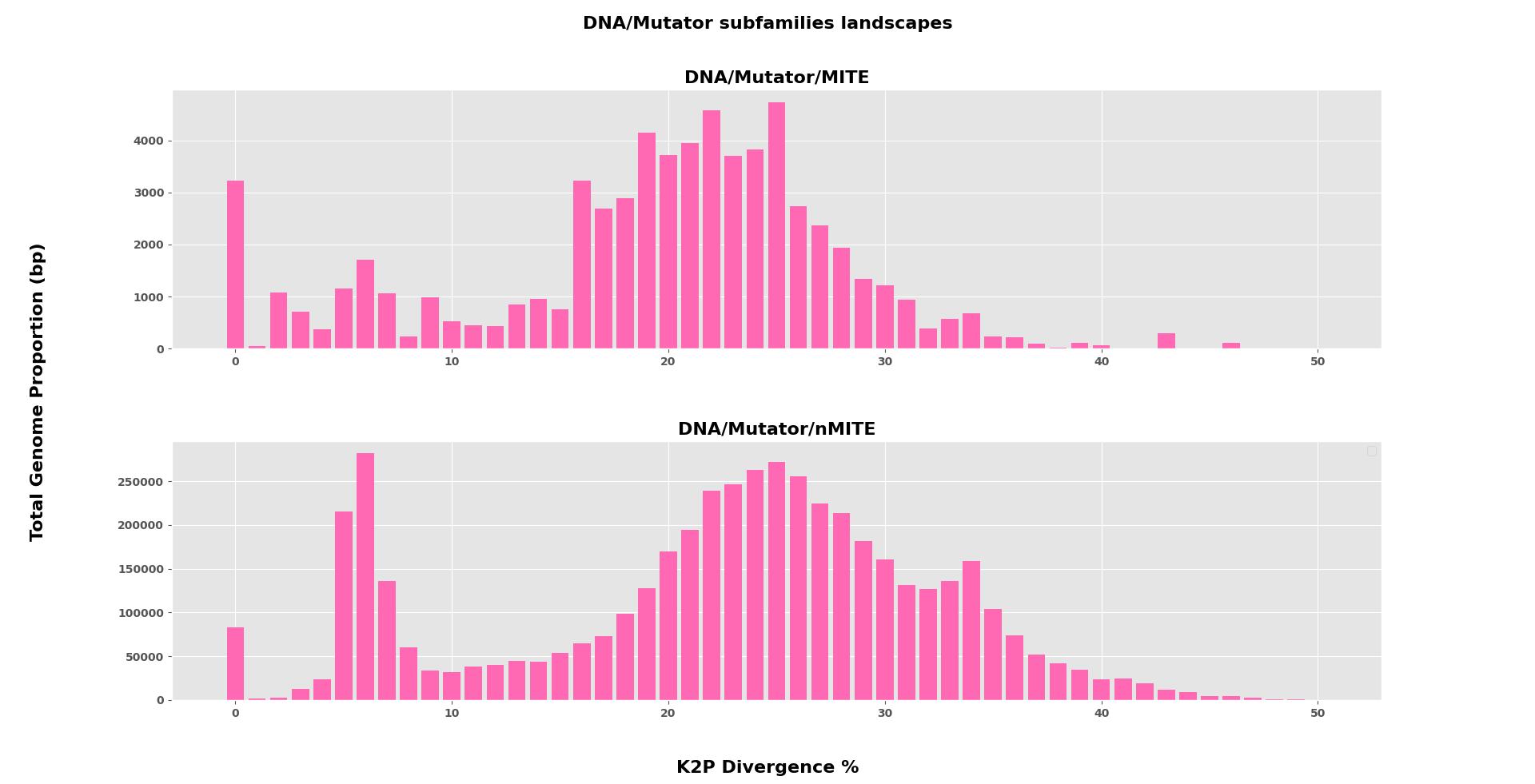


**Figure S8: DNA/Mutator subfamilies abundance and divergence repeat graphs.** The x-axis depicts the degree of divergence, while the y-axis represents the total genome proportion. The peaks on the graphs indicate the times of insertion of a particular subfamily in the genome. The right side of the graph indicates elements that were inserted earlier in the genome, whereas the left side indicates recent insertions.

| Anova |  | DF | SS | MS | F-value | P-value |
| --- | --- | --- | --- | --- | --- | --- |
|  | **Model** | **1** | **14321.81168** | **14321.81168** | **434.33938** | **0.00003** |
| Repeat (mb) | **Error** | **4** | **131.89512** | **32.97378** |  |  |
|  | **Total** | **5** | **14453.7068** |  |  |  |

**Table S1:** The table shows the results of the analysis of variance and Pearson's correlation between the total size of each chromosome and the size of the repeats present within it.

| Pearson’s correlation |  | Repeat size | Total size |
| --- | --- | --- | --- |
| Repeat size | **Pearson Corr** | **1** | **0.99543** |
|  | **p-value** | **--** | **0.00003** |
| Total size | **Pearson Corr** | **0.99543** | **1** |
|  | **p-value** | **0.00003** | **--** |

**p-value= 0.00003, r= 0.99**

**
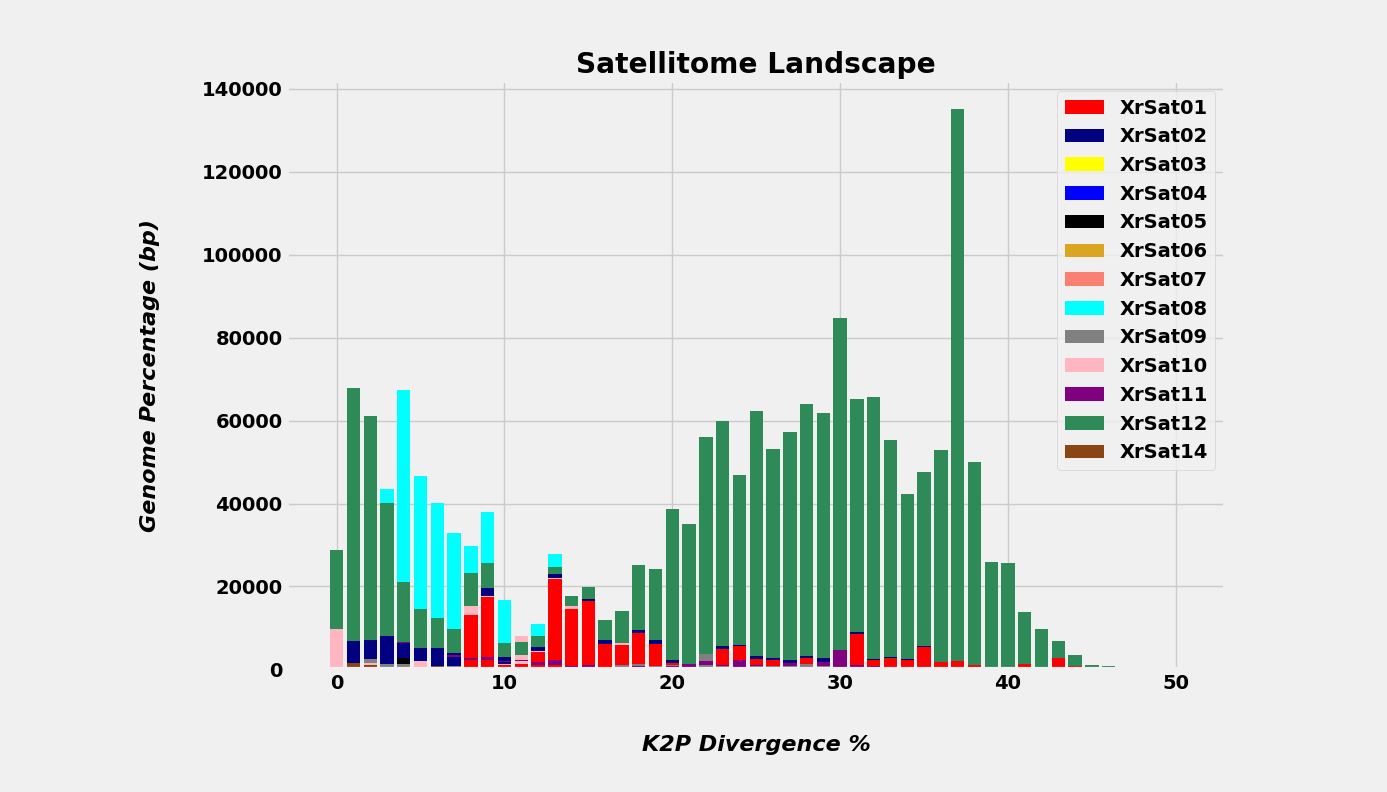
**

**Figure S9: Satellitome landscapes of *Xya riparia* genome.** The satellitome landscapes show the proportion of genome coverage for each satellite DNA family on the Y-axis. The X-axis shows the Kimura genetic distances between each satellite DNA family and their corresponding consensus sequence, ranging from 0% to 50% K2P.

**Table S2:** The table provides information about the divergence percentage, abundance percentage, and copy number of various satellite DNA families in *X. riparia*, which were estimated using RepeatMasker. Additionally, the table also lists the A+T percentage of each satellite DNA family.

| Families | Length (bp) | AbsLen (bp) | Kimura Divergence% | A+T% | Copy number | Abundance |
| --- | --- | --- | --- | --- | --- | --- |
| XrSat01 | 947 | 177271 | 18.88 | 54 | 18719.21 | 0.0118 |
| XrSat02 | 98 | 74599 | 7.61 | 67.5 | 76121.42 | 0.004 |
| XrSat03 | 29 | 4397 | 0.59 | 72.4 | 15162.06 | 0.0002 |
| XrSat04 | 185 | 25025 | 8.28 | 57.8 | 13527.02 | 0.001 |
| XrSat05 | 49 | 12686 | 2.46 | 65.3 | 25889.79 | 0.0008 |
| XrSat06 | 58 | 1493 | 0.63 | 50 | 2574.13 | 0.0009 |
| XrSat07 | 124 | 7134 | 3.1 | 54.8 | 5753.22 | 0.0004 |
| XrSat08 | 152 | 347362 | 6.04 | 58.6 | 228527.63 | 0.02 |
| XrSat09 | 127 | 36863 | 18.52 | 55.1 | 29025.98 | 0.002 |
| XrSat10 | 39 | 33606 | 5.95 | 61.5 | 86169.23 | 0.002 |
| XrSat11 | 173 | 43371 | 12.08 | 59 | 25069.94 | 0.002 |
| XrSat12 | 308 | 1456422 | 26.21 | 57.1 | 472864.28 | 0.09 |
| XrSat14 | 127 | 4044 | 3.21 | 59.1 | 3184.25 | 0.0002 |
| Avg/Total |  |  | **8.73** | **59.4** |  | **0.15** |

**Table S3:** This table contain the information about the differential expressed transposable elements log2FoldChange and p-values values.

| Elements_Name | Families | baseMean | log2FoldChange | lfcSE | stat | p-value |
| --- | --- | --- | --- | --- | --- | --- |
| Xya_R_00000167_ | LTR/Gypsy | 2.217 | 3.463 | 1.780 | 1.95 | 0.05 |
| Xya_R_00000200_INT | Bel-Pao | 131.197 | 1.373 | 0.495 | 2.77 | 0.01 |
| Xya_R_00000245 | LTR/Gypsy | 5.968 | 3.530 | 1.717 | 2.06 | 0.04 |
| Xya_R_00000252 | TcMar_nMITE | 4.782 | -2.415 | 1.114 | -2.17 | 0.03 |
| Xya_R_00000564_INT | LTR/Gypsy | 48.224 | -3.290 | 0.964 | -3.41 | 0.00 |
| Xya_R_00000637 | TcMar_nMITE | 10.580 | 1.366 | 0.710 | 1.92 | 0.05 |
| Xya_R_00000738 | PiggyBac | 82.434 | 1.918 | 0.643 | 2.98 | 0.00 |
| Xya_R_00000800_ | LTR/Gypsy | 9.095 | 1.643 | 0.812 | 2.02 | 0.04 |
| Xya_R_00000893 | DNA/Helitron | 13.626 | -2.293 | 1.061 | -2.16 | 0.03 |
| Xya_R_00000993_INT | MITE | 88.421 | -2.114 | 0.615 | -3.44 | 0.00 |
| Xya_R_00001074 | hAT_nMITE | 3.344 | -2.660 | 1.396 | -1.91 | 0.05 |
| Xya_R_00001095 | DNA/Helitron | 13.058 | -1.469 | 0.770 | -1.91 | 0.05 |
| Xya_R_00001306_INT | LTR | 13.348 | -3.030 | 1.030 | -2.94 | 0.00 |
| Xya_R_00001343 | DNA/Helitron | 188.199 | -1.070 | 0.481 | -2.22 | 0.03 |
| Xya_R_00001480 | hAT_nMITE | 13.191 | 1.607 | 0.675 | 2.38 | 0.02 |
| Xya_R_00001514 | hAT_nMITE | 24.134 | 1.870 | 0.823 | 2.27 | 0.02 |
| Xya_R_00001531_INT | LTR/Gypsy | 2.362 | 3.532 | 1.773 | 1.99 | 0.05 |
| Xya_R_00001533 | TcMar_nMITE | 28.637 | 3.613 | 0.874 | 4.13 | 0.00 |
| Xya_R_00001605 | Tc1_Mariner | 311.737 | -1.118 | 0.491 | -2.28 | 0.02 |
| Xya_R_00001878_ | LTR/Gypsy | 7.729 | 1.714 | 0.894 | 1.92 | 0.05 |
| Xya_R_00002058 | MITE | 9.045 | 2.973 | 0.943 | 3.15 | 0.00 |
| Xya_R_00002258_INT | LTR/Gypsy | 586.197 | 1.080 | 0.388 | 2.79 | 0.01 |
| Xya_R_00002404 | hAT_nMITE | 7.251 | -2.785 | 1.218 | -2.29 | 0.02 |
| Xya_R_00002506 | Penelope | 17.863 | 1.422 | 0.622 | 2.28 | 0.02 |
| Xya_R_00002566_INT | LTR/Gypsy | 3.474 | -4.261 | 1.609 | -2.65 | 0.01 |
| Xya_R_00002573 | CACTA_nMITE | 6.427 | -3.603 | 1.372 | -2.63 | 0.01 |
| Xya_R_00002583 | hAT/MITE | 4.562 | -2.629 | 1.145 | -2.30 | 0.02 |
| Xya_R_00002600 | hAT_nMITE | 31.443 | 1.161 | 0.439 | 2.64 | 0.01 |
| Xya_R_00002611 | nMITE | 5.351 | -2.040 | 1.060 | -1.92 | 0.05 |
| Xya_R_00002640 | nMITE | 90.078 | -1.160 | 0.544 | -2.13 | 0.03 |
| Xya_R_00002766 | TIR/hAT | 4.292 | 2.826 | 1.474 | 1.92 | 0.05 |
| Xya_R_00002799 | LTR/Gypsy | 38.469 | -1.122 | 0.560 | -2.01 | 0.04 |
| Xya_R_00002815 | Tc1_Mariner | 56.084 | -1.664 | 0.518 | -3.21 | 0.00 |
| Xya_R_00002873 | TcMar_nMITE | 10.219 | -2.062 | 0.936 | -2.20 | 0.03 |
| Xya_R_00003082 | MITE | 2.841 | -4.026 | 1.778 | -2.26 | 0.02 |
| Xya_R_00003110_INT | LTR | 13.090 | 1.587 | 0.808 | 1.96 | 0.05 |
| Xya_R_00003124_ | LTR/Gypsy | 17.595 | -1.400 | 0.631 | -2.22 | 0.03 |
| Xya_R_00003131_INT | LTR/Gypsy | 6.074 | -1.926 | 1.010 | -1.91 | 0.05 |
| Xya_R_00003193_INT | LTR/Gypsy | 2.008 | 4.390 | 2.296 | 1.91 | 0.05 |
| Xya_R_00003465_ | LTR/Gypsy | 17.203 | -3.455 | 0.904 | -3.82 | 0.00 |
| Xya_R_00003518 | Unknown | 7.469 | 2.073 | 0.974 | 2.13 | 0.03 |
| Xya_R_00003608_INT | LTR/Gypsy | 395.244 | 1.568 | 0.692 | 2.27 | 0.02 |
| Xya_R_00003633 | TcMar_nMITE | 80.544 | -1.982 | 0.539 | -3.68 | 0.00 |
| Xya_R_00003655 | TcMar_nMITE | 3.094 | 2.905 | 1.481 | 1.96 | 0.05 |
| Xya_R_00003696 | LINE | 32.955 | 1.604 | 0.678 | 2.36 | 0.02 |
| Xya_R_00003772 | MITE | 4.176 | -5.563 | 1.987 | -2.80 | 0.01 |
| Xya_R_00003909 | LTR/Copia | 53.874 | -2.210 | 0.636 | -3.48 | 0.00 |
| Xya_R_00004113 | nMITE | 5.790 | -4.074 | 1.729 | -2.36 | 0.02 |

**
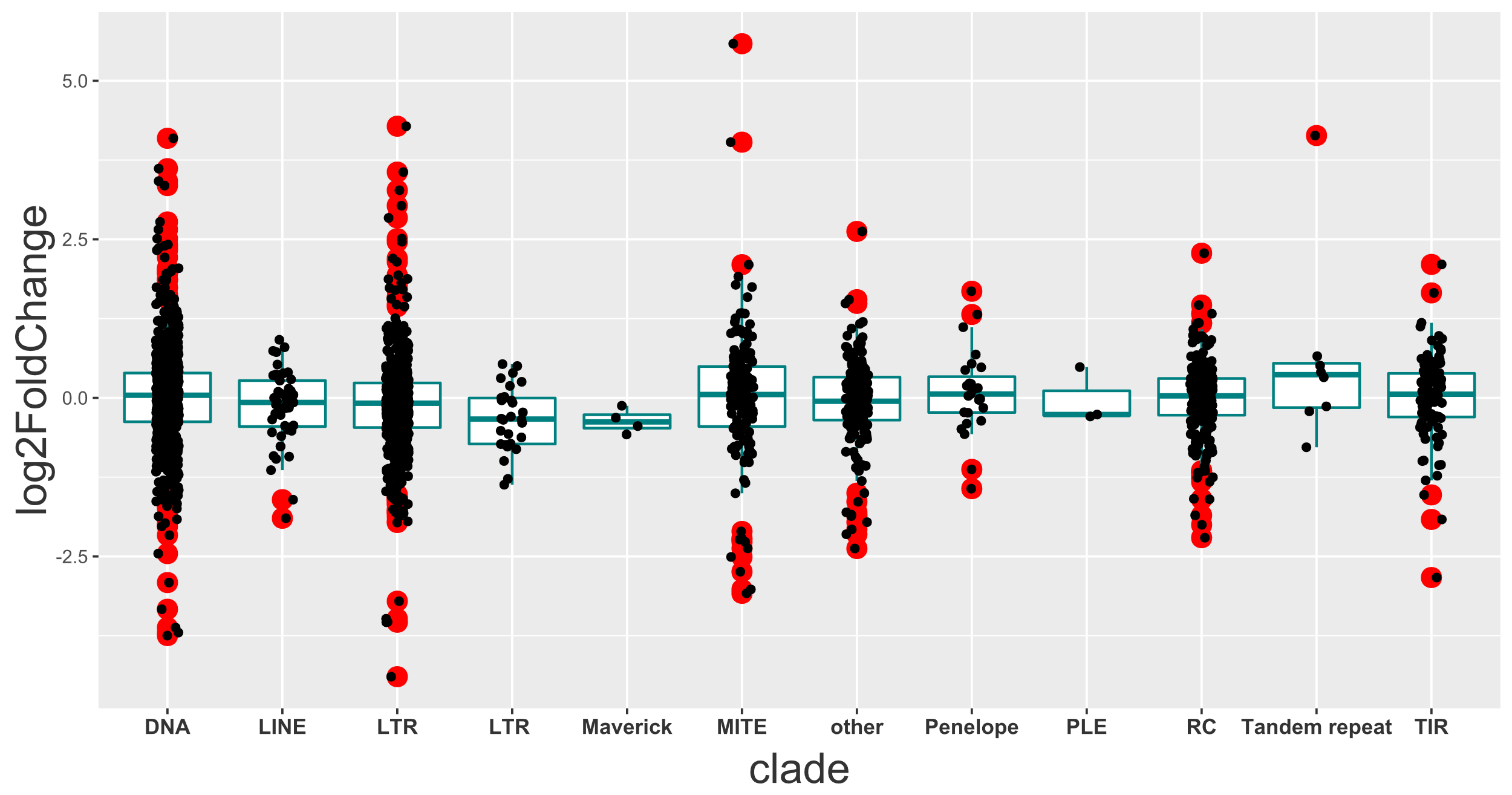
**

**Figure S10: A clade level log2FC boxplot of the TEs and differentially expressed TEs are represented as an outlier outside the whisker in the clade.**

**
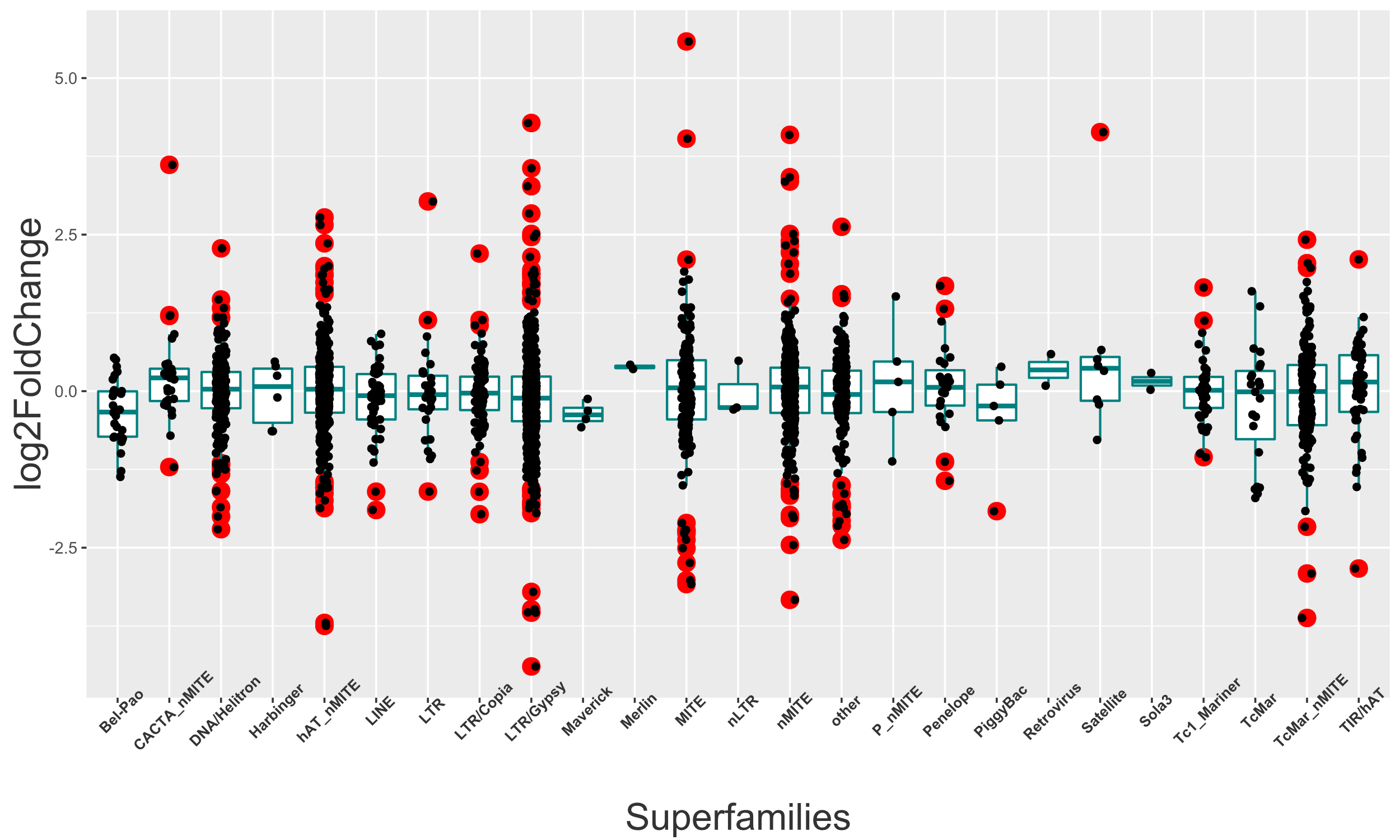
**

**Figure S11: A superfamily level log2FC boxplot of the TEs and differentially expressed TEs are represented as an outlier outside the whisker in the class.**

**
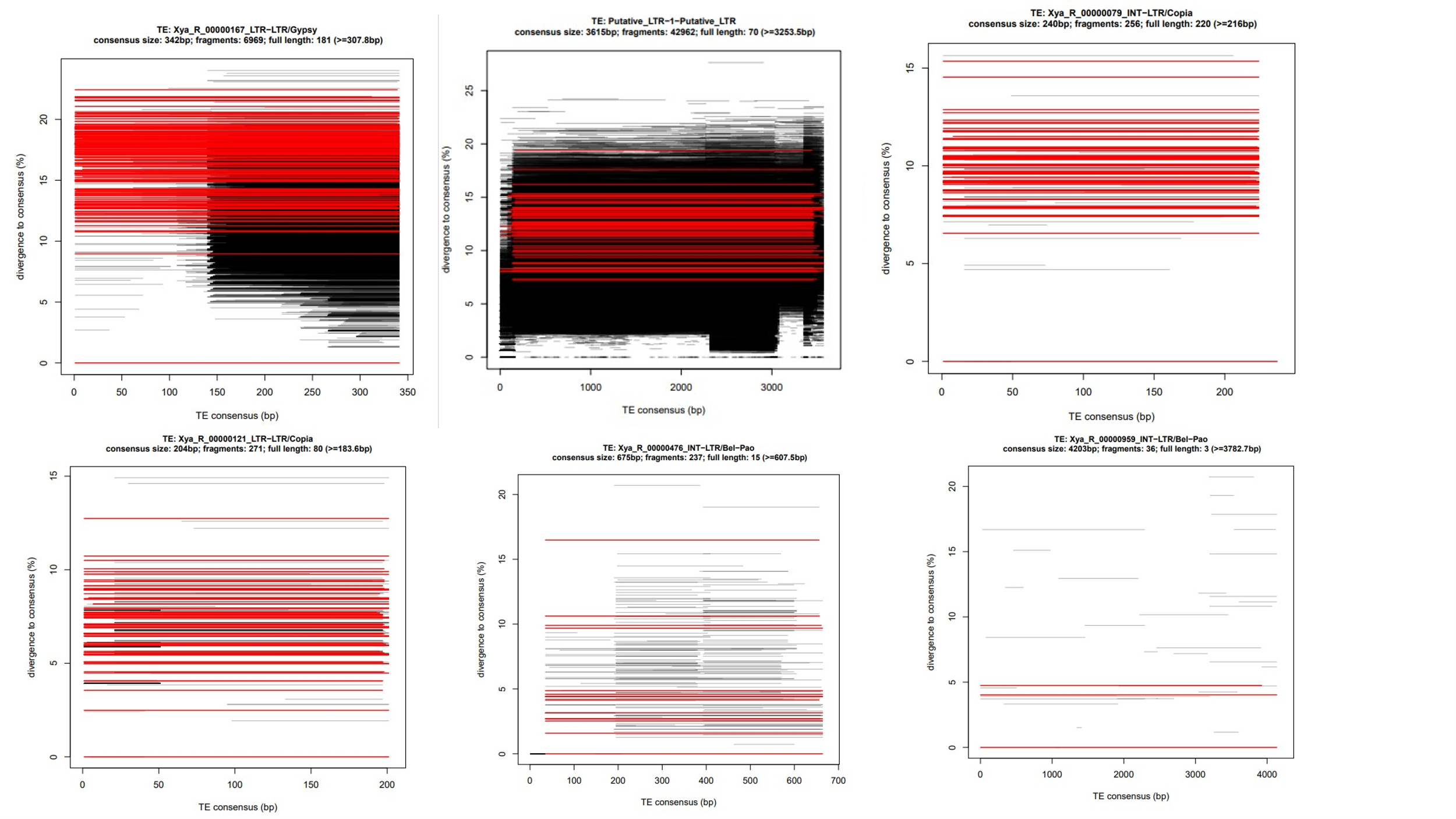
**

**Figure S12: The full-length copies of LTR/Gypsy, LTR/Copia, and LTR/Bel-pao identified within the genome of *X. riparia*.**

**Table S4: Table contains the information of total copy number, and full-length copies of each TEs superfamilies.**

| Repeat | Length | AbsLen (bp) | Avg.divergence | Abundance% | Copy number | Full-length |
| --- | --- | --- | --- | --- | --- | --- |
| ClassII/nMITE | 2701 | 87262838 | 19.2 | 5.817522533 | 3230760.385 | N/P |
| DNA/Harbinger | 558 | 1567258 | 11.3 | 0.104483867 | 280870.6093 | 50 |
| DNA/Helitron | 7417 | 108711542 | 19.7 | 7.247436133 | 1465707.725 | N/P |
| DNA/TcMar | 1527 | 42162652 | 17.3 | 2.810843467 | 2761142.895 | N/P |
| DNA/hAT | 2530 | 131950940 | 17.6 | 8.796729333 | 5215452.174 | N/P |
| LINE | 2741 | 40526430 | 23.5 | 2.701762 | 1478527.18 | N/P |
| LTR/Copia | 1470 | 24356174 | 10.2 | 1.623744933 | 1656882.585 | 312 |
| LTR/Bel-Pao | 1185 | 6193967 | 10.4 | 0.412931133 | 522697.6371 | 20 |
| LTR/Gypsy | 1018 | 171054211 | 14.3 | 11.40361407 | 16802967.68 | 325 |
| MITE | 325 | 19382712 | 12.3 | 1.2921808 | 5963911.385 | 66 |
| Maverick | 1896 | 3951977 | 15.1 | 0.263465133 | 208437.6055 | N/P |
| Penelope | 1127 | 2046381 | 23.3 | 0.1364254 | 181577.7285 | 15 |
| TIR | 3509 | 33792428 | 13.3 | 2.252828533 | 963021.6016 | N/P |
| Unknown | 2131 | 19621874 | 18.6 | 1.308124933 | 920782.4496 | N/P |
